# Functional characterization of Cullin-1-RING ubiquitin ligase (CRL1) complex in *Leishmania infantum*

**DOI:** 10.1371/journal.ppat.1012336

**Published:** 2024-07-17

**Authors:** Camila Rolemberg Santana Travaglini Berti de Correia, Caroline Torres, Ellen Gomes, Giovana Maffei Rodriguez, Wesley Klaysson Pereira Regatieri, Nayore Tamie Takamiya, Luana Aparecida Rogerio, Iran Malavazi, Marcelo Damário Gomes, Jeziel Dener Damasceno, Vitor Luiz da Silva, Marcos Antonio Fernandes de Oliveira, Marcelo Santos da Silva, Alessandro Silva Nascimento, Adriano Cappellazzo Coelho, Sandra Regina Maruyama, Felipe Roberti Teixeira

**Affiliations:** 1 Department of Genetics and Evolution, Federal University of São Carlos, São Carlos, Brazil; 2 Department of Biochemistry and Immunology, Ribeirão Preto Medical School, University of São Paulo, Ribeirão Preto, Brazil; 3 Institute of Infection, Immunity and Inflammation, University of Glasgow, Glasgow, United Kingdom; 4 Department of Biochemistry, Institute of Chemistry, University of São Paulo, São Paulo, Brazil; 5 Department of Chemical and Biological Sciences, Biosciences Institute, São Paulo State University (UNESP), Botucatu, Brazil; 6 São Carlos Institute of Physics, University of São Paulo, São Carlos, São Paulo, Brazil; 7 Department of Animal Biology, Institute of Biology, University of Campinas, Campinas, Brazil; University of Notre Dame, UNITED STATES OF AMERICA

## Abstract

Cullin-1-RING ubiquitin ligases (CRL1) or SCF1 (SKP1-CUL1-RBX1) E3 ubiquitin ligases are the largest and most extensively investigated class of E3 ligases in mammals that regulate fundamental processes, such as the cell cycle and proliferation. These enzymes are multiprotein complexes comprising SKP1, CUL1, RBX1, and an F-box protein that acts as a specificity factor by interacting with SKP1 through its F-box domain and recruiting substrates via other domains. E3 ligases are important players in the ubiquitination process, recognizing and transferring ubiquitin to substrates destined for degradation by proteasomes or processing by deubiquitinating enzymes. The ubiquitin-proteasome system (UPS) is the main regulator of intracellular proteolysis in eukaryotes and is required for parasites to alternate hosts in their life cycles, resulting in successful parasitism. *Leishmania* UPS is poorly investigated, and CRL1 in *L*. *infantum*, the causative agent of visceral leishmaniasis in Latin America, is yet to be described. Here, we show that the *L*. *infantum* genes LINF_110018100 (SKP1-like protein), LINF_240029100 (cullin-like protein-like protein), and LINF_210005300 (ring-box protein 1 –putative) form a LinfCRL1 complex structurally similar to the *H*. *sapiens* CRL1. Mass spectrometry analysis of the LinfSkp1 and LinfCul1 interactomes revealed proteins involved in several intracellular processes, including six F-box proteins known as F-box-like proteins (Flp) (data are available via ProteomeXchange with identifier PXD051961). The interaction of LinfFlp 1–6 with LinfSkp1 was confirmed, and using *in vitro* ubiquitination assays, we demonstrated the function of the LinfCRL1(Flp1) complex to transfer ubiquitin. We also found that *LinfSKP1* and *LinfRBX1* knockouts resulted in nonviable *L*. *infantum* lineages, whereas *LinfCUL1* was involved in parasite growth and rosette formation. Finally, our results suggest that LinfCul1 regulates the S phase progression and possibly the transition between the late S to G2 phase in *L*. *infantum*. Thus, a new class of E3 ubiquitin ligases has been described in *L*. *infantum* with functions related to various parasitic processes that may serve as prospective targets for leishmaniasis treatment.

## Author summary

In eukaryotes, intracellular proteolysis is mostly performed by the ubiquitin-proteasome system (UPS), which regulates numerous cellular functions by ubiquitinating regulatory proteins that may be degraded by the proteasome. Substrate recruitment and ubiquitination are mediated by E3 ubiquitin ligases. CRL1 (Cullin-1-RING ligases) are the largest and most researched family of E3 proteins in eukaryotes, comprising SKP1, Cullin1, RBX1, and F-box proteins. The life cycle and infection of *Leishmania* depends on the UPS, and the role of CRL1 in these parasites has not been studied. Here, we identified CRL1 proteins in *L*. *infantum* and demonstrated the assembly of LinfCRL1, which shares a three-dimensional structure with the *H*. *sapiens* complex, suggesting a functional relationship. A set of LinfSkp1 and LinfCul1 protein partners were found to be associated with several cellular processes, indicating their pleiotropic effects. Six F-box proteins were found interacting with LinfSkp1 and were designated F-box-like proteins (Flp). Flp1 assembly in an active LinfCRL1(Flp1) complex capable of ubiquitin transfer. Our study demonstrated the critical role of *LinfSKP1* and *LinfRBX1* in the parasite, whereas knockout of *LinfCUL1* resulted in impaired proliferation and duplication, along with the stimulation of rosette formation. In addition, LinfCul1 regulates *L*. *infantum* cell cycle progression. Overall, we characterized a novel E3 ubiquitin ligase class that could potentially be targeted by antileishmanial drugs.

## Introduction

*Leishmania infantum* and *L*. *donovani* are the etiological agents of visceral leishmaniasis (VL), the most severe form of leishmaniasis, with fatality rates of up to 95% if not treated. One billion people worldwide live in regions endemic to leishmaniasis, with an estimated 50,000–90,000 new VL cases per year globally, most of which occur in Brazil, East Africa, and India [1]. *Leishmania* has a dixenous lifestyle that comprises an extracellular stage, the promastigote form, which replicates in the gut of sandflies over two replicative phases that consist of various differentiation stages, and an intracellular stage, the amastigote form, which replicates within mammalian macrophages [2,3]. This intricate life cycle underscores the importance of understanding the molecular mechanisms governing their survival and replication under diverse conditions. As *Leishmania* infections continue to pose a serious health problem worldwide, it is critical to unravel the molecular machinery of the parasite. In this regard, investigation of the ubiquitin-proteasome system (UPS) has emerged as a promising avenue, considering its pivotal role in regulating various cellular processes. The UPS orchestrates targeted protein degradation, regulating important aspects of parasite biology [4–8].

Proteasomes are multi-subunit, multicatalytic proteases responsible for most of intracellular proteolysis in eukaryotes [9]. *L*. *mexicana* proteasome is similar to proteasomes from other eukaryotes [10] and its inhibition has been explored as a potential target for chemotherapy of leishmaniasis [5,7]. Lactacystin, a proteasome-specific inhibitor, suppressed the growth of *L*. *infantum* promastigotes *in vitro* and impaired the survival of amastigotes in infected host cells when the promastigotes were previously treated with the inhibitor [11]. These findings demonstrate that the UPS is required for parasite replication and amastigote survival within the host cells.

The UPS comprises of three classes of enzymes responsible for protein ubiquitination and the 26S proteasome. Initially, ubiquitin-activating enzyme (E1) catalyzes ubiquitin activation in an ATP-dependent reaction. Furthermore, activated ubiquitin (Ub) is transferred to the ubiquitin-conjugating enzyme (E2), and ubiquitin ligases (E3), which specifically interact with substrates, recruit E2-Ub, culminating in ubiquitination of the substrate [12], which can be reversed by deubiquitinating enzymes (DUBs). E3 ubiquitin ligases are classified into two main classes based on their E2 interaction domains: HECT (Homologous to E6AP Carboxyl Terminus), which directly catalyzes the covalent attachment of ubiquitin to substrate proteins, and RING type (Really Interesting New Gene), which is characterized by their RING or RING-like (e.g., U-box) catalytic domain that promotes Ub transfer from an E2-Ub to the substrate [13]. There are approximately 30 E3 HECT-like ligases in mammals and more than 600 RING-like E3 ligases have been predicted in the human genome [14]. RING-type E3 ubiquitin ligase members can function as monomers, dimers, and heterodimers or can be multi-subunits based on the Cullin protein, which includes Cullin-1 RING ubiquitin ligases (CRL1s), also called the SCF1 complex (SKP1, CUL1, RBX1, and F-box) [13]. CUL1 is a scaffold protein for the RING box protein 1 (RBX1) at the C-terminus and SKP1 at the N-terminus. It is an adapter for F-box proteins (FBP) that uses the F-box motif to interact with SKP1 and other domains to recruit their substrates for the CRL1 complex. Thus, FBPs are specificity factors of CRL1 or SCF1 E3 ubiquitin-ligase complexes [15–17].

Two E1 enzymes have been described in *L*. *major* [18], 13 E2 enzymes, 79 E3 ubiquitin ligases [19], and 20 deubiquitinating enzymes (DUBs) have been described in *L*. *mexicana* [20]. The E2 enzymes UBC1/CDC34, UBC2, UEV1, and E3 HECT2 are required for the differentiation of promastigotes to amastigotes. The E1 enzyme UBA1b, E2 enzymes UBC9, UBC14 and E3 enzymes HECT7 and HECT11 are required for proliferation during infection in mice [19]. In *L*. *major*, Kin13 protein is degraded by the parasite UPS, and its endogenous levels depend on the cell cycle [21]. In *L*. *donovani*, degradation of pteridine reductase 1 (PTR1) has been suggested to be related to the peptide ^63^QADLSNVAK^71^ and lysine residue 156 (K156) as probable ubiquitination site, suggesting that the proteasome is the main regulator of its level in the parasite [22]. Three proteasome inhibitors, MG132, lactacystin, and epoxomycin, lead to the accumulation of methionine adenosyltransferase (MAT) protein in parasites. Additionally, MAT was conjugated with ubiquitin molecules, suggesting that the UPS was responsible for the degradation of this substrate [23].

CRLs E3 ubiquitin ligases are responsible for eukaryotic cell cycle regulation via ubiquitination of different substrates [24]. Despite their well-established roles in cellular processes in various organisms, the involvement of CRLs in *Leishmania* remains unexplored. In this study, we unveiled the identity of CRL1 components in *L*. *infantum* and identified by mass spectrometry the LinfSkp1 and LinfCul1 protein partners involved in different cellular processes, including the UPS. We showed that CRL1 is assembled in *L*. *infantum* in association with six F-box proteins, collectively known as F-box-like proteins (Flp). Through *in vitro* ubiquitination assays, we provided compelling evidence demonstrating the ability of LinfCRL1(Flp1) to transfer ubiquitin. Finally, gene knockouts of LinfCRL1 components revealed that *LinfSKP1* and *LinfRBX1* are critical genes, and that *LinfCUL1* is involved in cellular proliferation, rosette formation and cell cycle regulation in *L*. *infantum* promastigotes.

## Results

### Identification of CRL1 genes in *Leishmania* spp

**Fig 1:**
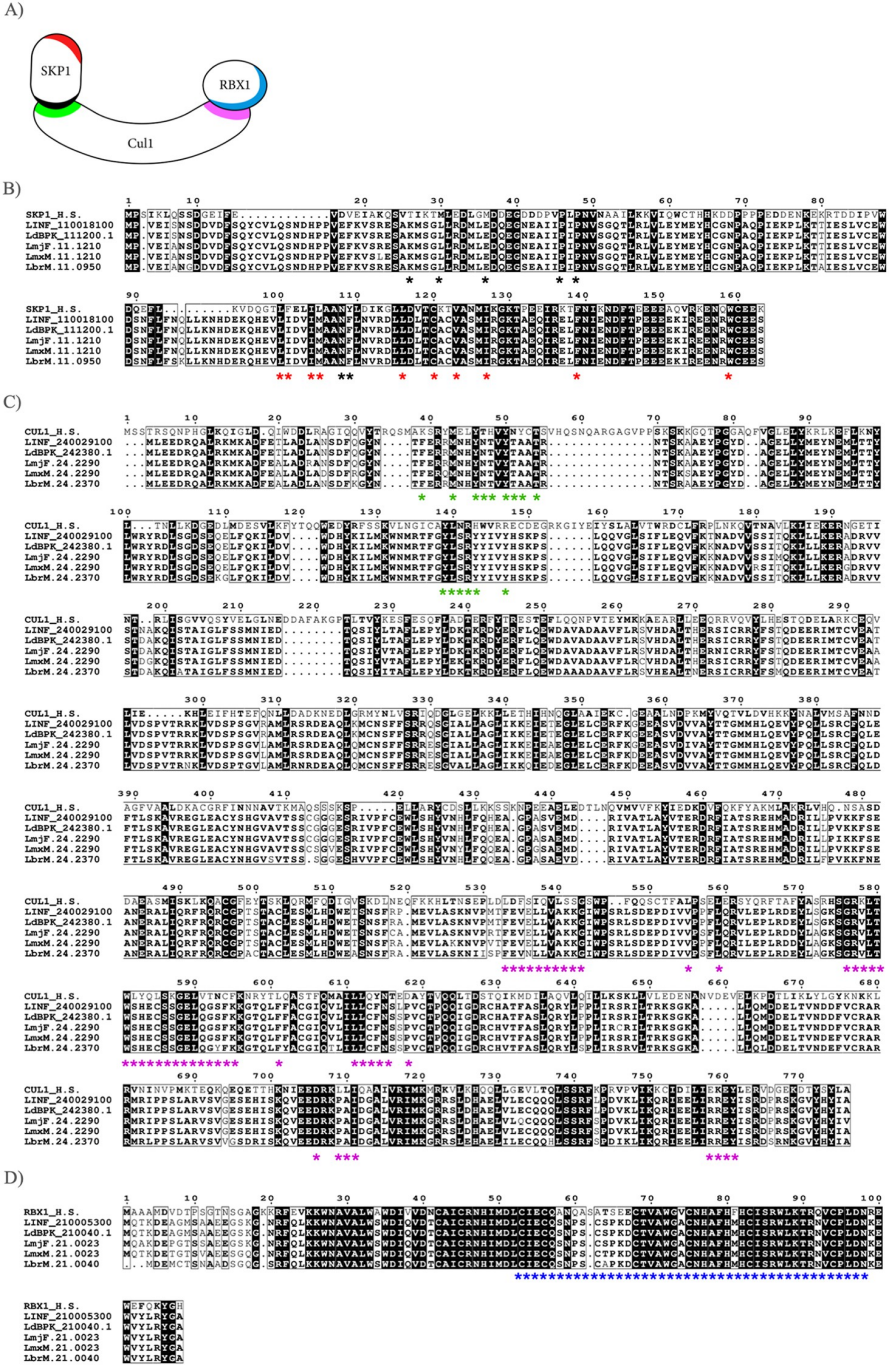
Multiple sequence alignment (MSA) of human SKP1, CUL1, and RBX1 proteins with their respective *Leishmania* protein orthologs. A) Schematic illustration of the CRL complex, highlighting the regions where its components interact. B) SKP1: *H*. *sapiens* (Uniprot: P63208), *L*. *infantum* (LINF_110018100), *L*. *donovani* (LdBPK_111200.1), *L*. *major* (LmjF.11.1210), *L*. *mexicana* (LmxM.11.1210) and *L*. *braziliensis* (LbrM.11.0950). Red asterisks indicate residues that interact with the F-box domain and black asterisks indicate SKP1 residues that interact with Cullin 1. C) CUL1: *H*. *sapiens* (Uniprot: Q13616), *L*. *infantum* (LINF_240029100), *L*. *donovani* (LdBPK_242380.1), *L*. *major* (LmjF.24.2290), *L*. *mexicana* (LmxM.24.2290) and *L*. *braziliensis* (LbrM.24.2370). Green and purple asterisks indicate the CUL1 residues that interact with SKP1 and RBX1, respectively. D) RBX1: *H*. *sapiens* (Uniprot: P62877), *L*. *infantum* (LINF_210005300), *L*. *donovani* (LdBPK_210040.1), *L*. *major* (LmjF.21.0023), *L*. *mexicana* (LmxM.21.0023) and *L*. *braziliensis* (LbrM.21.0040). Blue asterisks indicate the RING domain of RBX1. MSAs were performed using the MUSCLE method [27] and visualized using the ESPript 3.0 [28]. The highlighted boxes indicate the conserved regions across the protein sequences.

The CRL1 complex comprises CUL1 as a scaffold protein to allow interaction with SKP1 at its N-terminus and RBX1 at its C-terminus. SKP1, in turn, interacts with an F-box protein at different binding interfaces that interact with CUL1 (Fig 1A). Orthologous genes corresponding to *H*. *sapiens SKP1*, *CUL1*, and *RBX1* were searched in *L*. *infantum* genome using TriTrypDB resources [25]. The genes LINF_110018100 (SKP1-like protein), LINF_240029100 (cullin-like protein-like protein), and LINF_210005300 (ring-box protein 1 –putative) were retrieved, and their translated sequences displayed 63, 21 and 67% amino acid identity with their respective human orthologs, respectively. These orthologs were also identified in other *Leishmania* species, such as *L*. *donovani*, *L*. *major*, *L*. *mexicana*, and *L*. *braziliensis*, with a similar identity degree observed in *L*. *infantum*, demonstrating that these proteins are conserved in these parasites (Fig 1B–1D).

Guided by the crystallographic structure of human CRL1 [17,26], we identified crucial residues for complex CRL1 assembly in *L*. *infantum* (color pattern shown in Fig 1A). Conserved amino acid residues of SKP1/CUL1 and CUL1/RBX1 interaction regions were observed in *Leishmania* (highlighted by asterisks, Fig 1B–1D). Although LinfCul1 has a low amino acid identity (21%) with its human ortholog, essential residues for its interaction with LinfSkp1 and LinfRbx1 are highly conserved (Fig 1C, green and purple asterisks, respectively). LinfSkp1 residues that are important for interaction with the F-box domain and LinfCul1 are also conserved (Fig 1B, red and black asterisks, respectively). The RING box domain of LinfRbx1 (residues 53–98), which mediates interaction with E2, is also highly conserved in *Leishmania* (Fig 1D, blue asterisks). These findings indicate that the residues responsible for the assembly of LinfCRL1 are conserved in *Leishmania*, suggesting its presence in these parasites.

### Phylogenetic analysis of CRL1 genes in *Leishmania*

Since the genome of *Leishmania* spp. are largely conserved, it is plausible to assume that genes that code for proteins of the CRL1 complex exist in other species of the *Leishmania* genus. Thus, we expanded our *in silico* search of the CRL1 complex to include 12 species. Blastx searches in TriTrypDB using human *SKP1*, *CUL1*, and *RBX1* sequences retrieved the orthologous of *L*. *braziliensis*, *L*. *panamensis*, *L*. *amazonensis*, *L*. *mexicana*, *L*. *donovani*, *L*. *infantum*, *L*. *aethiopica*, *L*. *tropica*, *L*. *major*, *L*. *gerbilli*, *L*. *arabica*, and *L*. *turanica*. We assessed phylogenetic relationships in each reconstructed tree for each member of the CRL1 complex.

**Fig 2:**
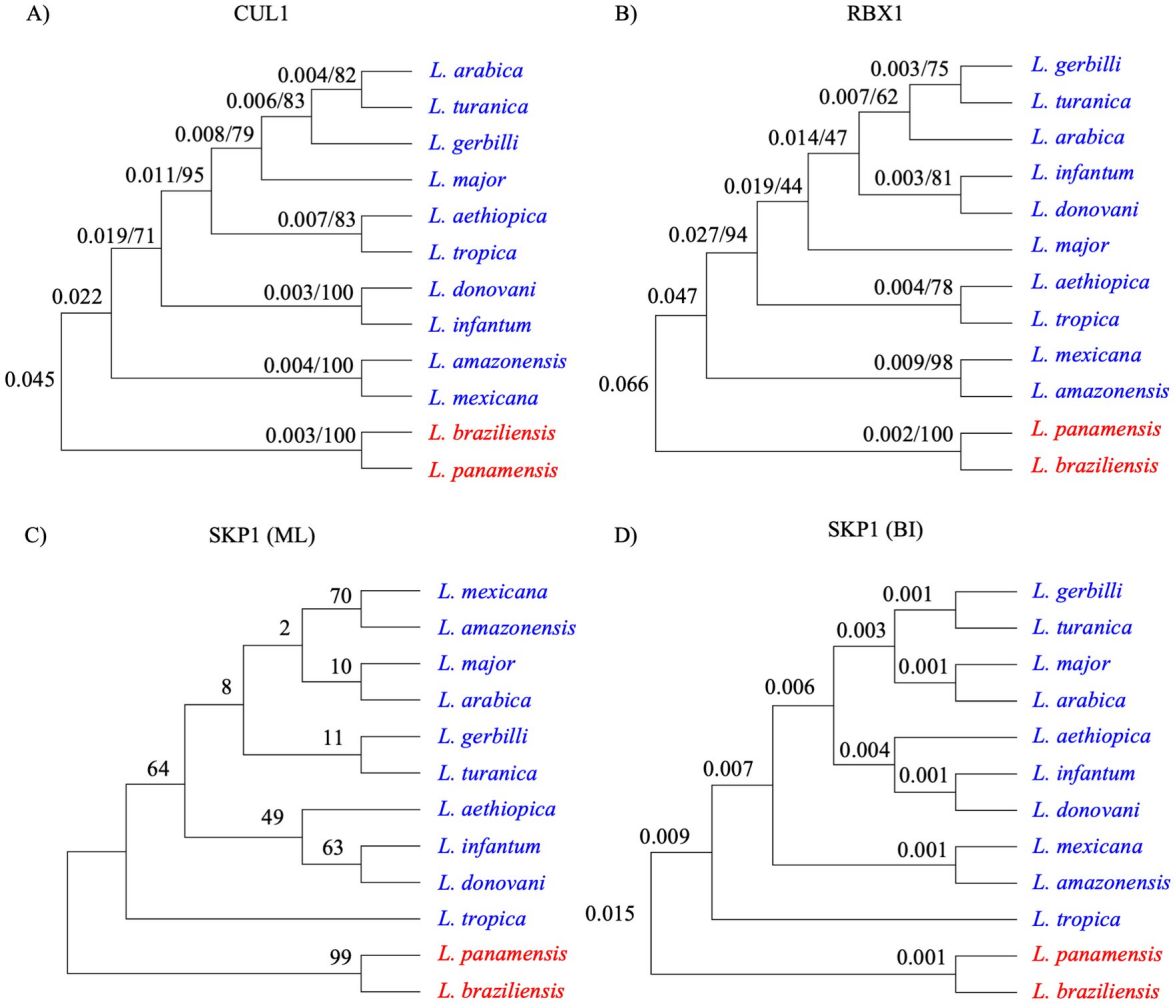
Phylogenetic trees of CRL1 complex proteins from *Leishmania*. A) CUL1, B) RBX1 and SKP1 (C-D) cladograms built with 12 orthologs of *Leishmania* spp. (accession numbers in S1 Table). The proteins were aligned using the MUSCLE method [27] and trees were reconstructed using ML and BI methods. A total of 183, 744, and 106 positions for SKP1, CUL1, and RBX, respectively, were considered for the final dataset. ML analysis was performed using MEGA11 software [32], while BI analysis was performed using BEAST software [33]. The numbers next to the branches represent the statistical support of phylogenetic inference displayed by method side by side, IB (probability) / ML (boostrap), for CUL1 (A) and RBX1 (B); for SKP1 cladograms are displayed according to the method of inference (C and D). Species within *Leishmania* and *Viannia* subgenera are indicated in blue and red font, respectively.

Multiple sequence alignment (MSA) obtained through MUSCLE for each protein was verified for the amino acid substitution model, in which LG [29], JTT [30] and JTT with gamma distribution were the most suitable for SKP1, CUL1, and RBX1, respectively. Maximum Likelihood (ML) and Bayesian Inference (BI) methods were then used to reconstruct the phylogenetic trees for each protein group. Overall, the phylogenetic relationships among species based on the reconstructions of the orthologous SKP1, CUL1, and RBX1 proteins (Fig 2) resemble phylogenomic analysis using approximately 5,600 orthologous proteins of trypanosomatids [31]. As expected, in all phylogenies, *L*. *infantum* forms a clade with *L*. *donovani* within the *Leishmania* subgenus, as does *L*. *mexicana* with *L*. *amazonensis*, and *L*. *braziliensis* with *L*. *panamensis* within the *Viannia* subgenus.

Evolutionary relationships with highest statistical support (bootstrap > 70% and probability < 0.05) were given remarkably to CUL1 phylogeny (Fig 2A) and RBX1 phylogeny (Fig 2B). Bayesian-inferred phylogenies for CUL1 and RBX1 were displayed in S1 Fig For SKP1, most of the branches were not well-supported in phylogeny inferred by the ML method (Fig 2C), except for the clade formed by species of *Viannia* subgenus (*L*. *braziliensis* and *L*. *panamensis*). In turn, Bayesian inference reconstructed a statistically robust tree for SKP1 phylogeny (Fig 2D), including a better placement of the clade formed by *L*. *mexicana* and *L*. *amazonensis*, which was inconsistent in the ML-inferred tree. Overall, the phylogeny of the large multidomain CUL1 protein (796AA) proved to be highly conserved, most likely because of its key role as a scaffold in the CRL1 assembly complex.

### Protein interaction assays with LinfSkp1, LinfCul1 and LinfRbx1

**Fig 3:**
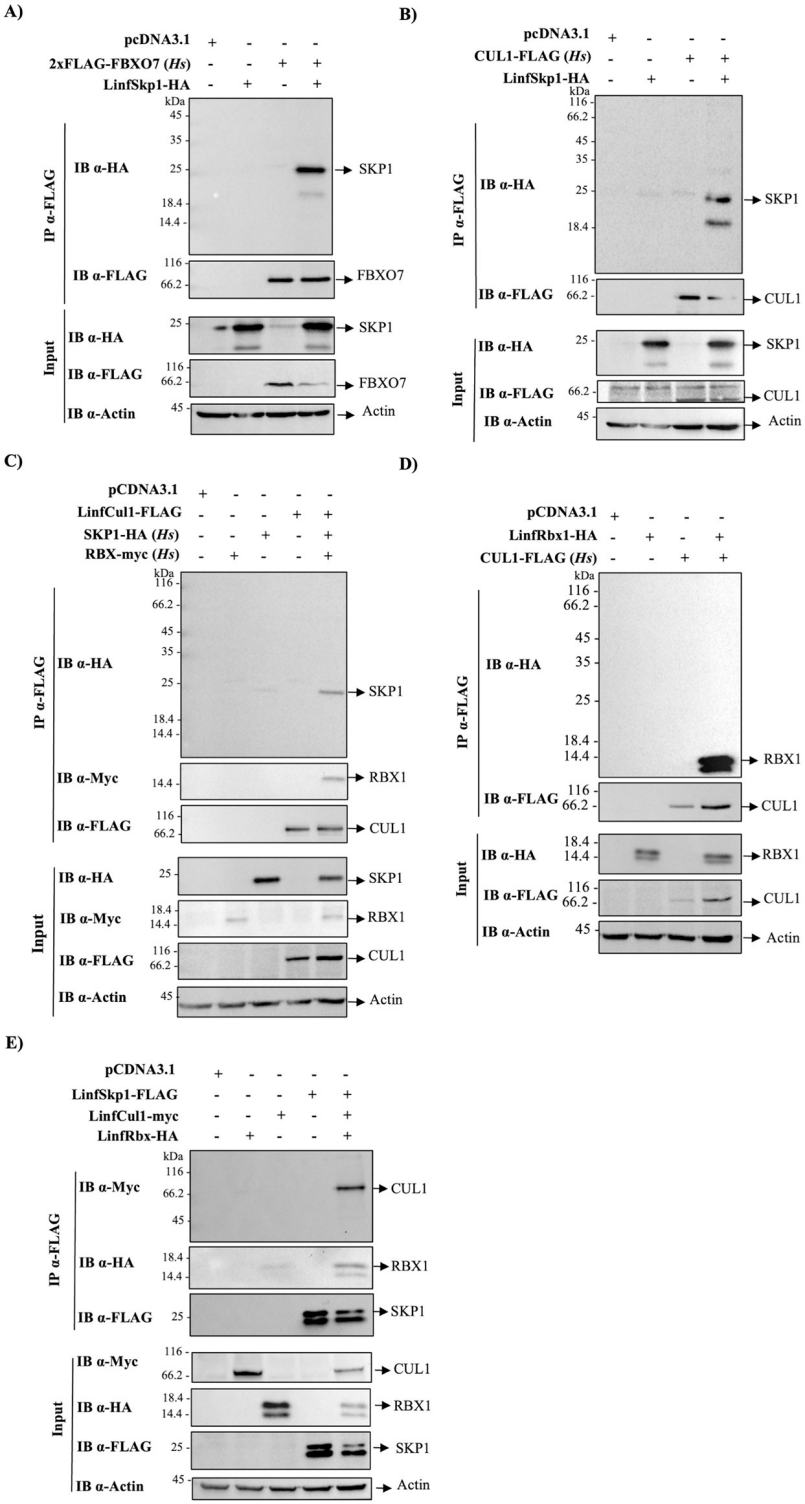
Interaction of human and *L*. *infantum* CRL1 proteins. HEK293T cells were transfected with the indicated plasmids and cellular extracts were subjected to immunoprecipitation with agarose anti-FLAG beads. Co-immunoprecipitation of FBXO7 *(Hs)* and LinfSkp1 (A), CUL1 *(Hs)* and LinfSkp1 (B), LinfCul1 and SKP1 *(Hs)* and RBX1 *(Hs)* (C) and LinfRbx1 and human CUL1 *(Hs)* (D) and LinfSkp1, LinfCul1 and LinfRbx1 (E). The pcDNA3.1 plasmid is an empty vector utilized as a negative control. Antibodies used are indicated. All assays were performed in duplicate.

To investigate whether the conserved interaction regions of the *L*. *infantum* CRL1 (LinfCRL1) components are sufficient to promote the assembly of this complex, we evaluated by co-immunoprecipitation (co-IP) the interaction among the parasite proteins and their respective partners of *H*. *sapiens (Hs)*. Initially, HEK293T cells were co-transfected with LinfSkp1-HA and a human protein containing the F-box domain (2xFLAG-FBXO7) *(Hs)*, which interacts with SKP1*(Hs)* [34]. After co-IP, we observed that these proteins interacted, suggesting that the interaction region of LinfSkp1 with the F-box domain is conserved (Fig 3A). Similarly, LinfSkp1-HA and CUL1-FLAG *(Hs)* interacted, indicating that LinfSkp1 also possesses a conserved interaction domain with CUL1 (Fig 3B) in addition to the F-box interaction domain (Fig 3A). To evaluate the LinfCul1 interaction regions with SKP1 and RBX1, HEK293T cells were co-transfected with LinfCul1-FLAG, SKP1-HA *(Hs)*, and RBX1-myc *(Hs)*. Co-IP results demonstrated that LinfCul1 interacted with both proteins (Fig 3C). Lastly, we assessed whether LinfRBX1 interacts with CUL1 *(Hs)* (Fig 3D). The results demonstrated that both proteins interact, reinforcing the significant conservation of the interaction domain between these proteins, as shown by *in silico* analyses. Finally, to assess whether LinfCRL1 proteins can assemble a CRL1 complex, co-IP among LinfSkp1-FLAG, LinfCul1-myc, and LinfRbx1-HA was performed, and the results showed that these proteins were co-eluted, demonstrating that they assemble a LinfCRL1 complex in *L*. *infantum* (Fig 3E).

### LinfCRL1 shares structural similarities with the *H*. *sapiens* complex

To gain insight into the structure of LinfCRL1 compared to *H*. *sapiens*, we searched in AlphaFold database [35,36] for the structures of proteins encoded by the genes LINF_110018100 (LinfSkp1), LINF_240029100 (LinfCul1), and LINF_210005300 (LinfRbx1) of *L*. *infantum* JPCM5 and compared them individually to their human orthologs (S2 Fig). These findings demonstrated a comparable folding pattern of these proteins. The root-mean-square distance (RMSD), a quantitative measure assessing the disparity between corresponding atoms in two structures, confirmed a high similarity between SKP1 proteins with an RMSD value of 1.7 Å. For identical structures, the RMSD is 0, and values within the range of 0–2 Å indicate a high percentage of identity [37]. The RMSD values for CUL1 and RBX1 were 4.8 Å and 7 Å, respectively, indicating that although these proteins are not identical, they exhibit notable structural resemblances.

**Fig 4:**
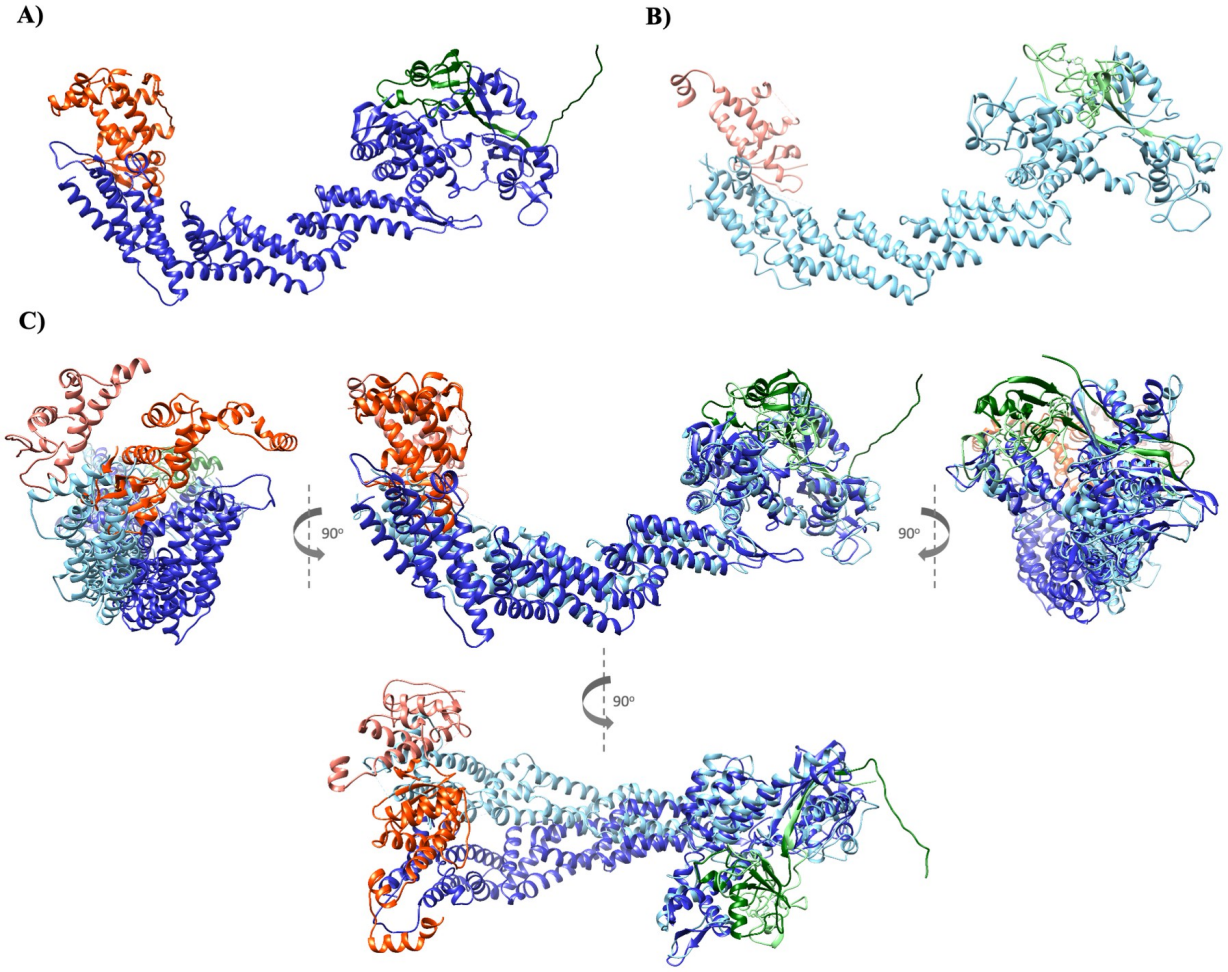
Structure of CRL1 complexes from *L*. *infantum* and *H*. *sapiens* generated by AlphaFold. A) LinfCRL1 with LinfSkp1 (red), LinfCul1 (blue), and LinfRbx1 (green). B) *H*. *sapiens* CRL1 with SKP1 protein (light red), CUL1 (light blue), and RBX1 (light green). C) Superposition of the *L*. *infantum* and *H*. *sapiens* CRL1 complexes at different angles.

To analyze the LinfCRL1 complex structure, AlphaFold was used to assemble these proteins in a complex using AlphaFold multimer [38]. Interestingly, structural models generated showed highly similar topology and folding profiles for both complexes (Fig 4A and 4B). LinfCul1 function as a scaffold for the LinfCRL1 complex, interacting with LinfSkp1 at the N-terminus and LinfRbx1 at the C-terminus. The upper view of the complex superimposition from both organisms (Fig 4C, bottom image) revealed a twist in LinfCul1 compared with *H*. *sapiens* CUL1. This could be explained by the low identity with the human protein in its central region (Fig 1B) and the lower confidence of its N-terminus observed in the structure predicted by AlphaFold (S2 Fig). However, the model returned the same structural pattern for the binding of LinfRbx1 to LinfCul1 at the C-terminus ends as the *H*. *sapiens* complex (Fig 4C).

Reliability of the LinfCRL1 complex was evaluated using AF2 prediction. The model obtained for the complex contained 1,133 residues and was predicted with an average pLDDT of 81,62, indicating a complex model with good confidence for individual structures and the complex [38]. The complex was colored by AF2 using a pLDDT scale, which indicated that most portions of the model were highly reliable, with pLDDT over 80.0, and the less reliable fragments were restricted to mobile fragments at the C- and N-termini of individual proteins (S3 Fig). Thus, the LinfCRL1 complex model not only demonstrated significant structural resemblance to the CRL1 complex from *H*. *sapiens*, but also exhibited high reliability in each component and the predicted complex, indicating a conserved function in substrate ubiquitination.

### Interactome of LinfSkp1 and LinfCul1

**Fig 5:**
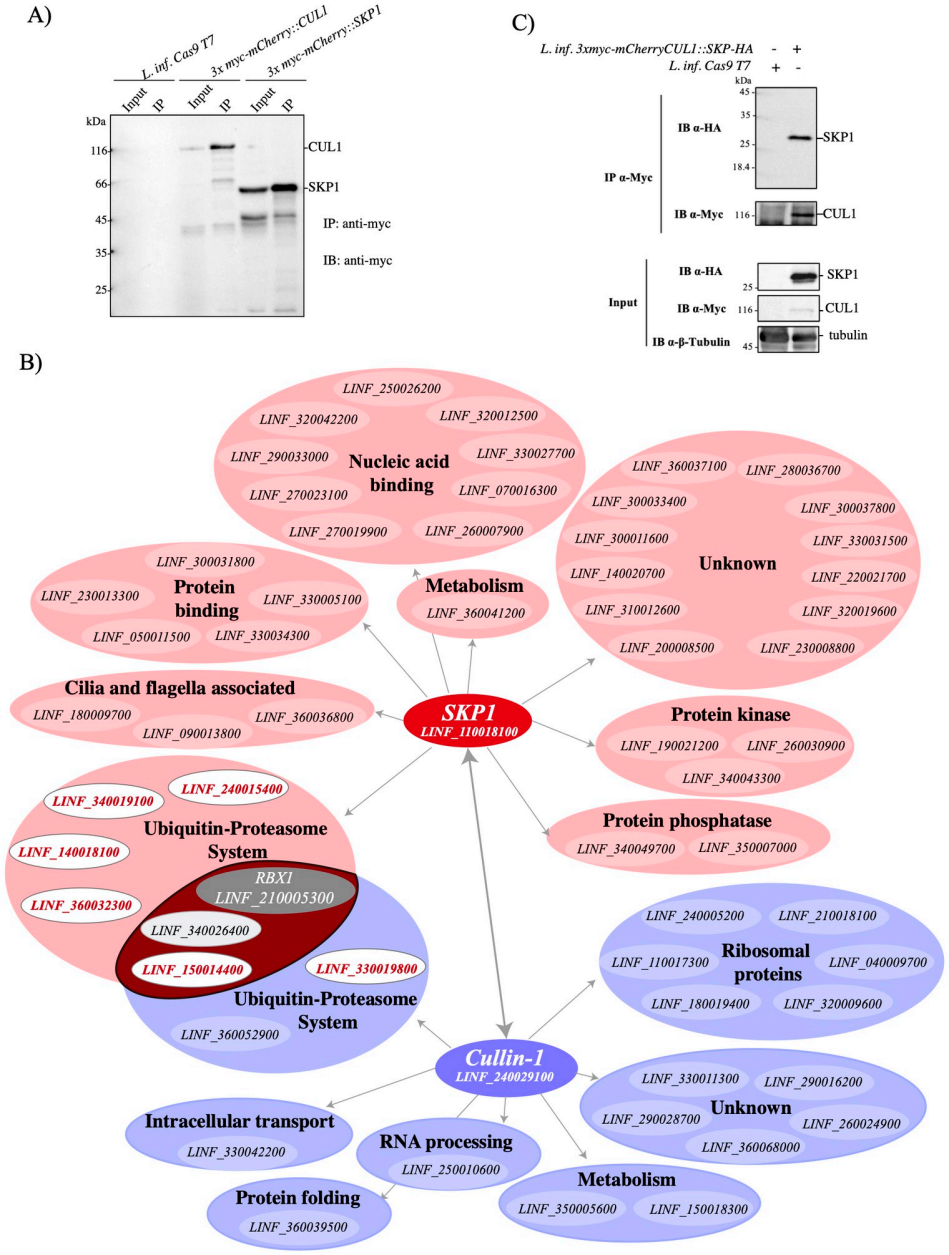
The interactomes of LinfSkp1 and LinfCul1. A) *L*. *infantum* Cas9 T7, *L*. *infantum* Cas9 T7::3x *myc-mCherry*::*LinfCUL1* or *L*. *infantum* Cas9 T7:: *3x myc-mCherry*::*LinfSKP1* strains were lysed and subjected to immunoprecipitation with anti-myc agarose resin. The total extract (input) and eluates were resolved by 12% SDS-PAGE and analyzed by western blotting with anti-myc. B) The interactome of LinfSkp1 and LinfCul1 with a network of interactions with protein partners identified by mass spectrometry, where F-box proteins are highlighted in red. C) Interaction of HA-LinfSkp1 and 3x myc-mCherry-LinfCul1 in *L*. *infantum* cell lines expressing both proteins with fusion tags. The total extract (input) and eluates from IP with anti-myc were resolved by SDS-PAGE and analyzed by western blotting using the indicated antibodies.

To confirm the assembly of LinfCRL1, the interactome of LinfSkp1 and LinfCul1 was carried out. CRISPR-Cas9 technology was used to generate N-terminally tagged *3x myc-mCherry*::*LinfCUL1* or *3x myc-mCherry*::*LinfSKP1* lines in a transgenic line of *L*. *infantum* HU-UFS14 expressing Cas9 and T7 RNA polymerase as previously described [39]. The transgenic lines were confirmed by diagnostic PCR using specific primers targeting the integrated cassette (S4A Fig). These lines showed no difference in their respective duplication rates when compared to the *L*. *infantum*-Cas9 T7 parental strain or *L*. *infantum* wild-type parasite (S4B Fig). Proteins from these strains were extracted and subjected to immunoprecipitation (IP) with anti-myc conjugated beads, and the results showed that 3xmyc-mCherry-LinfSkp1 and 3xmyc-mCherry-LinfCul1 were expressed and purified (Fig 5A).

To obtain the interactome of LinfSkp1, LinfCul1, the parental strain Cas9 T7, and *L*. *infantum* strains were subjected to large-scale IP with anti-myc beads. After washing, the beads were directly digested by trypsin, aiming to increase peptides recovery, and were processed for mass spectrometry analysis [40]. The proteins identified in the negative control were excluded from the LinfSkp1 and LinfCul1 interactomes, and a total of 43 unique proteins were identified in the LinfSkp1 interactome and 22 in the LinfCul1 interactome (Fig 5B and S2 Table). Based on the number of unique peptides, LinfCul1 had the highest score in the LinfSkp1 interactome, and LinfRbx1 was identified. LinfSkp1 was the second most abundant protein in the LinfCul1 interactome, which also identified LinfRbx1 (S2 Table and Fig 5B). These results confirm the assembly of the LinfCRL1 complex. To validate the interaction between LinfSkp1 and LinfCul1, a transgenic line expressing HA-LinfSkp1 and 3x myc-mCherry-LinfCul1 was generated (S4C Fig). Promastigote parasites were lysed and subjected to IP with anti-myc beads, and the eluate confirmed the interaction between both proteins in *L*. *infantum* (Fig 5C).

In addition to SKP1, CUL1, and RBX1, active CRLs comprise an F-box protein that binds to SKP1 and recruits the substrate for ubiquitination [11,12]. Interestingly, five F-box proteins were identified in the LinfSkp1 interactome and two were identified in the LinfCul1 interactome (Fig 5B). Other proteins identified in the LinfSkp1 and LinfCul1 interactomes were UPS members (9 proteins), including a ubiquitin-like protein, as well as proteins related to different cellular processes, such as phosphatases (2), kinases (3), nucleic acid binding (9), cilium and flagellum-related proteins (2), ribosomal proteins (6), metabolism-related proteins (1), RNA processing (1), intracellular transport (1), and proteins of unknown function (18) (Fig 5B). These results confirmed the presence of an active CRL1 complex in *L*. *infantum* and further supported the role of LinfSkp1 and LinfCul1 in fundamental parasitic processes.

### F-box proteins associated with functional LinfCRL1 complex

**Fig 6:**
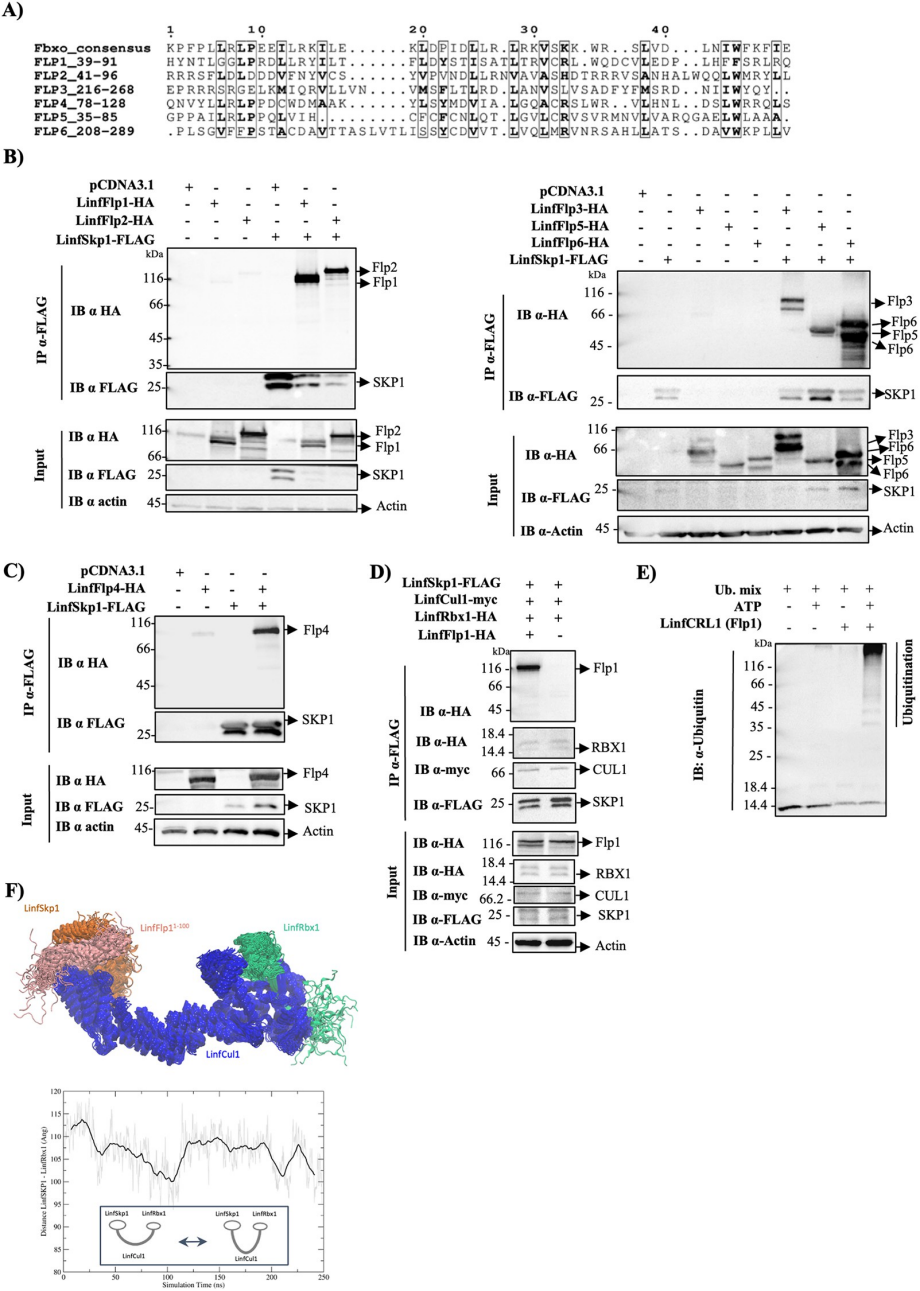
Characterization of LinfFlps and molecular dynamics of LinfCRL1(Flp1^1–100^). A) F-box consensus sequencing alignment with the F-box domain of Flps 1–6, where boxes represent the most conserved amino acid residues. B) To validate the LinfSkp1 interactome, HEK293T cells were transfected with the indicated plasmids, and cellular extracts were subjected to immunoprecipitation with anti-FLAG agarose beads. Co-immunoprecipitation of LinfSkp1 with LinfFlp1, LinfFlp2, LinfFlp3, LinfFlp5, and LinfFlp6 (B), and LinfFlp4 (C). LinfCRL1(Flp1) was purified (D) and its activity was evaluated using an *in vitro* ubiquitination assay (E). F) Molecular dynamic simulation of LinfCRL1(Flp1^1–100^), where LinfCul1 is shown in blue cartoon, LinfSkp1 is shown in orange cartoon while LinfRbx1 and LinfFlp1^1–100^ are shown in green and pink cartoon, respectively. The pcDNA3.1 plasmid is an empty vector utilized as a negative control. Antibodies used are shown. These assays were performed in duplicate.

F-box proteins contain a conserved motif of 48 amino acid residues for interaction with SKP1 and a substrate interaction domain, ensuring the specificity of ubiquitination by CRL1 complexes. In humans, there are approximately 69 F-box proteins that play a central role in regulating various cellular processes such as proliferation, cell cycle progression, transcription, and apoptosis [41]. Consequently, dysregulation of the ubiquitination process due to overexpression, low expression, or mutation of these proteins leads to several human diseases, including cancer [24]. The search for F-box domains in *L*. *infantum* proteins using InterPro (IPR036047—F-box-like domain superfamily) or ortholog genes in Uniprot and TritrypDB identified 11 proteins with an F-box domain in this parasite, of which six were identified in the LinfSkp1 and LinfCul1 interactomes (Table 1). The alignment of the F-box consensus sequence [41] with these six proteins, identified a conservation of the main residues involved in the interaction with SKP1 (Fig 6A).

**Table 1:** List of 11 F-box like proteins identified in the *L*. *infantum* genome, of which 6 were found in the interactome of LinfSkp1 or LinfCul1.

Gene	Uniprot	Name	Interactome	IP validated	F-box-like protein (Flp)
LINF_240015400	A4I0T9 or A0A6L0XEF5	F-box domain-containing protein	LinfSkp1	X	Flp1
LINF_150014400	A4HWG7 or A0A6L0WUC5	hypothetical protein - conserved	LinfSkp1/LinfCul1	X	Flp2
LINF_140018100	A0A6L0WRD5	FYVE-type domain-containing protein	LinfSkp1	X	Flp3
LINF_330019800	A4I8Y7 or A0A6L0XXY7	Hypothetical_protein_-_conserved	LinfCul1/interacted with LinfSkp1	X	Flp4
LINF_340019100	A4I9T3 or A0A6L0XNV2	Hypothetical_protein_-_conserved	LinfSkp1	X	Flp5
LINF_360032300	A4ICF5 or A0A6L0Y1Z0	Hypothetical_protein_-_conserved	LinfSkp1	X	Flp6
LINF_350013800	A0A6L0XPW7	Hypothetical_protein_-_conserved			Flp7
LINF_090021400	A4HU61 or A0A6L0WNL8	Hypothetical_protein_-__conserved			Flp8
LINF_210025500	A4HZJ5 or A0A6L0XMW7	F-box domain-containing protein			Flp9
LINF_300017500	A4I5F0 or A0A6L0XVF5	JmjC domain-containing protein			Flp10
LINF_350013800	A4IAY8	Uncharacterized protein			Flp11

Although F-box proteins have been described in trypanosome genomes [42], no studies on this protein group have demonstrated its function in *Leishmania* spp. Therefore, to elucidate the function of genes termed hypothetical or unknown in *Leishmania* spp. genomes, we proposed classifying the F-box proteins identified in this study as F-box-like proteins (Flp) (Table 1). To validate the interactions of LinfFlp1, LinfFlp2, LinfFlp3, LinfFlp5 and LinfFlp6 with LinfSkp1, co-IP assays were performed in HEK293T cells transfected with plasmids encoding these genes. The eluates revealed the interaction of these LinfFlps with LinfSkp1 (Fig 6B), confirming the interactome results. The proteins LinfFlp1 (LINF_240015400) and LinfFlp2 (LINF_150014400) were identified as the second and third proteins in the LinfSkp1 interactome, respectively, with the highest number of sequenced peptides, only behind LinfCul1, which is a partner of LinfSkp1 in the CRL1 complex (Fig 5C). Since the number of observed peptides has been utilized to ascertain the abundance of F-box proteins associated with CRL complexes [43], our findings indicate a strong association of LinfFlp1 and LinfFlp2 with LinfSkp1, suggesting a function of LinfCRL1(Flp1) and LinfCRL1(Flp2) in *L*. *infantum*.

LinfFlp2 was identified in the LinfCul1 interactome, with the highest number of peptides, followed by LinfSkp1 (S2 Table). We speculate that this interaction is a result of LinfCul1 interaction with LinfSkp1, which indirectly brings LinfFlp2 from the functional complex. In fact, LinfFlp4, which only appeared in the LinfCul1 interactome, did not directly interact with LinfCul1, as demonstrated by the co-IP results (S4D Fig). However, LinfFlp4 interacted with LinfSkp1 in the co-IP assay (Fig 6C), thus reinforcing the assumption of an indirect interaction between LinfFlp2 and LinfCul1 in its interactome.

To fully demonstrate the functional assembly of the active LinfCRL1(Flp1) complex, we developed a co-IP assay using anti-FLAG beads in HEK293T cells transfected with plasmids encoding LinfSkp1-FLAG, LinfCul1-myc, LinfRbx1-HA, and LinfFlp1-HA. The eluates confirmed the presence of the LinfCRL1(Flp1) complex with all components (Fig 6D). To further evaluate whether this complex was active, an *in vitro* ubiquitination assay was performed using human reagents E1(UBE1), E2 (UbcH5c/UBE2D3), and ubiquitin. We observed that LinfCRL1(Flp1) formed a polyubiquitination smear, confirming the activity of the purified complex (Fig 6E).

To visualize the F-box motif of Linf Flp1 interacting with LinfSkp1, Molecular Dynamics (MD) simulations of LinfCRL1(Flp1^1–100^) were performed. The trajectory frames obtained in the MD were clustered in the UCSF Chimera [44], and a representative frame of the most populated cluster is shown (Fig 6F). The complex assembly is structurally conserved compared to that of *H*. *sapiens* (PDB ID:1LDK) [17] and Flp1–100 stably interacts with LinfSkp1. Interestingly, the MD simulation showed an intrinsic mobility of the LinfCul1 protein in a bending movement that brings LinfSkp1 and LinfRbx1 closer to each other. This movement can be observed by monitoring the distance between the centers of mass of the two proteins (Fig 6F, bottom and S1 Video). This analysis indicates that, at approximately 100 ns of simulation time, the distance between these proteins decreased to approximately 95 Å, increasing again to approximately 110 Å afterwards. This bending movement of LinfCul1 has not been previously described in its *H*. *sapiens* ortholog.

### LinfCul1 is involved in proliferation and rosette formation

To evaluate whether LinfCRL1 proteins are essential for *L*. *infantum* promastigote survival, both alleles of the genes LINF_110018100 (SKP1), LINF_210005300 (RBX1), and LINF_240029100 (Cullin1) were simultaneously targeted for deletion using CRISPR-Cas9 technology [39]. Only the Cullin1 knockout strain (Δ*cul1*) was viable, indicating that *LinfSKP1* and *LinfRBX1* are potentially essential for *L*. *infantum*. In *T*. *brucei*, knockdown TbSkp1 and TbRbx1 by RNAi reduced the parasite proliferation rate, but both strains remained viable. Conversely, the knockout of TbCul1 did not lead to any observable effect, suggesting that other Cullins might perform its function in the parasite [45]. The *SKP1* and *RBX1* genes in *H*. *sapiens* are associated with ubiquitination by CRL1 complexes, and these findings highlight the importance of this complex in the parasite.

**Fig 7:**
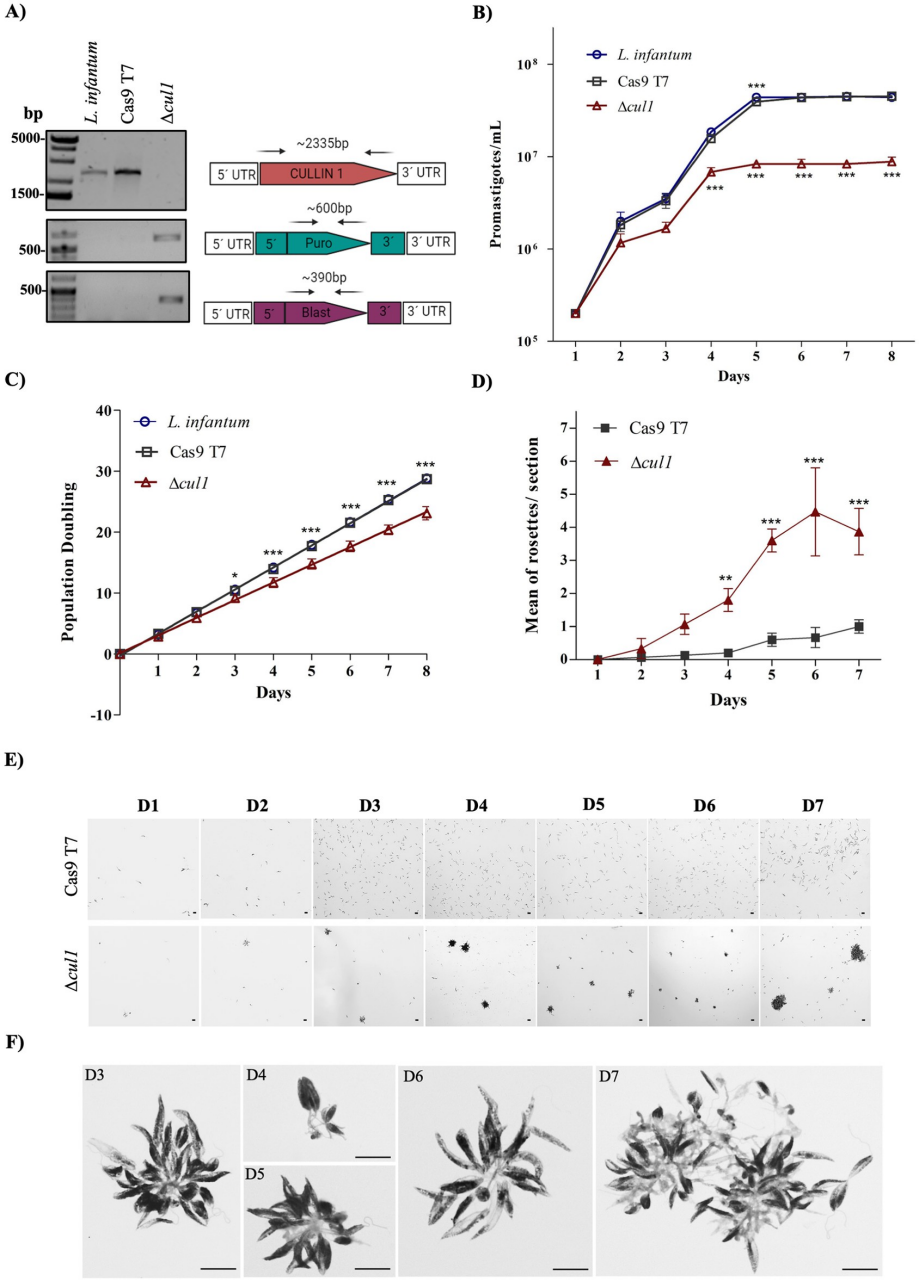
Effects of *LinfCUL1* knockout on *L*. *infantum* promastigotes. A) Amplicons from *L*. *infantum* HU-UFS14, Cas9 T7, and Δ*cul1* lines generated by PCR with the primers shown in the right diagram, which depicts the *LinfCUL1* gene *locus* and primer annealing sites (arrows). B) Growth curves of wild-type *L*. *infantum*, the Cas9 T7 transgenic line, and *Δcul1*. Cell counts were performed every 24 h for 8 days. C) Cell proliferation rate analysis by population doubling over eight days of culture between the control and Δ*cul1* strains. D) Comparison of rosette formation between the control and Δ*cul1* strains over an 8-day culture period. E) Promastigote culture images of the parental and Δ*cul1* strains showing rosette formation and growth throughout the culture period. Images were captured using a 20X objective. F) Smear of the parental and *L*. *infantum* Δ*cul1* promastigote cultures after panoptic staining. Representative rosettes of each period were observed under an optical microscope at 100X objective. B-C) Graphs were generated using GraphPad Prism v5.0, and statistical significance was determined using a Two-Way ANOVA test (*p<0.05; ***p<0.001).

*L*. *infantum* Δ*cul1* was validated by PCR, which was used to determine the presence of the *CUL1* gene coding sequence and the respective integration of the drug resistance markers (Fig 7A). Growth curve analysis of the Δ*cul1* lineage compared with wild-type *L*. *infantum* and Cas9 T7 revealed that the *LinfCUL1* knockout promoted a reduction in the growth rate from the fourth day onward (Fig 7B). To evaluate the cell growth rate more accurately, we performed a population doubling (PD) assay, in which we measured the cell line capacity to double over the course of culture days. In this assay, we assessed whether the proliferative potential of the cell line changed after cell passage. Interestingly, from the third passage forward, we found a divergence in the PD curves of the Δ*cul1* promastigotes compared to the respective controls, which was exacerbated over subsequent passages (Fig 7C). These findings indicated that *LinfCUL1* is directly related to cell proliferation and affects the duplication capacity of the parasite.

Observations of cell cultures revealed a profile consisting of a high number of rosettes in the Δ*cul1* line compared to the wild-type strain, especially from the fourth day of growth (Fig 7D). Intriguingly, this structure coincided with the time point at which the proliferation rate decreased, indicating that they may be related. The images demonstrated an increase in both the number and size of rosettes over the course of culture (Fig 7E), as well as intertwining flagella and the presence of fusion bodies (Fig 7F). Our findings indicated that *LinfCUL1* likely regulates the formation of these structures. Nevertheless, it is unclear whether this effect is associated with the non-canonical function of LinfCul1 or with the LinfCRL1 complex.

### LinfCul1 regulates the *L*. *infantum* cell cycle

The primary mechanism of cell cycle progression in eukaryotic cells is related to the successive activation of cyclin-dependent kinases (CDKs), which are mainly triggered by proteasomal degradation of their cyclin partners and kinase inhibitors (CKIs). Two eukaryotic E3 ubiquitin ligases, the anaphase-promoting complex (APC) and CRL1, are responsible for the ubiquitination of these regulators, thus regulating the progression of the cell cycle [24,46]. Cul1 is the scaffold protein for the CRL1 complex that interacts with four different F-box proteins (FBXW7, βTrCP, SKP2, and Cyclin F), and mediates the ubiquitination and degradation of cell cycle key regulators, such as Myc, Cyclin F, CDK inhibitors (p21, p27 and p57), claspin, WEE1, EMI1 and REST [46]. Here, we observed a reduction of cell proliferation in *L*. *infantum* Δ*cul1* compared to the parental cell lineage and carried out cell cycle analysis of both cell lineages to evaluate the function of LinfCul1 in this cellular process.

**Fig 8:**
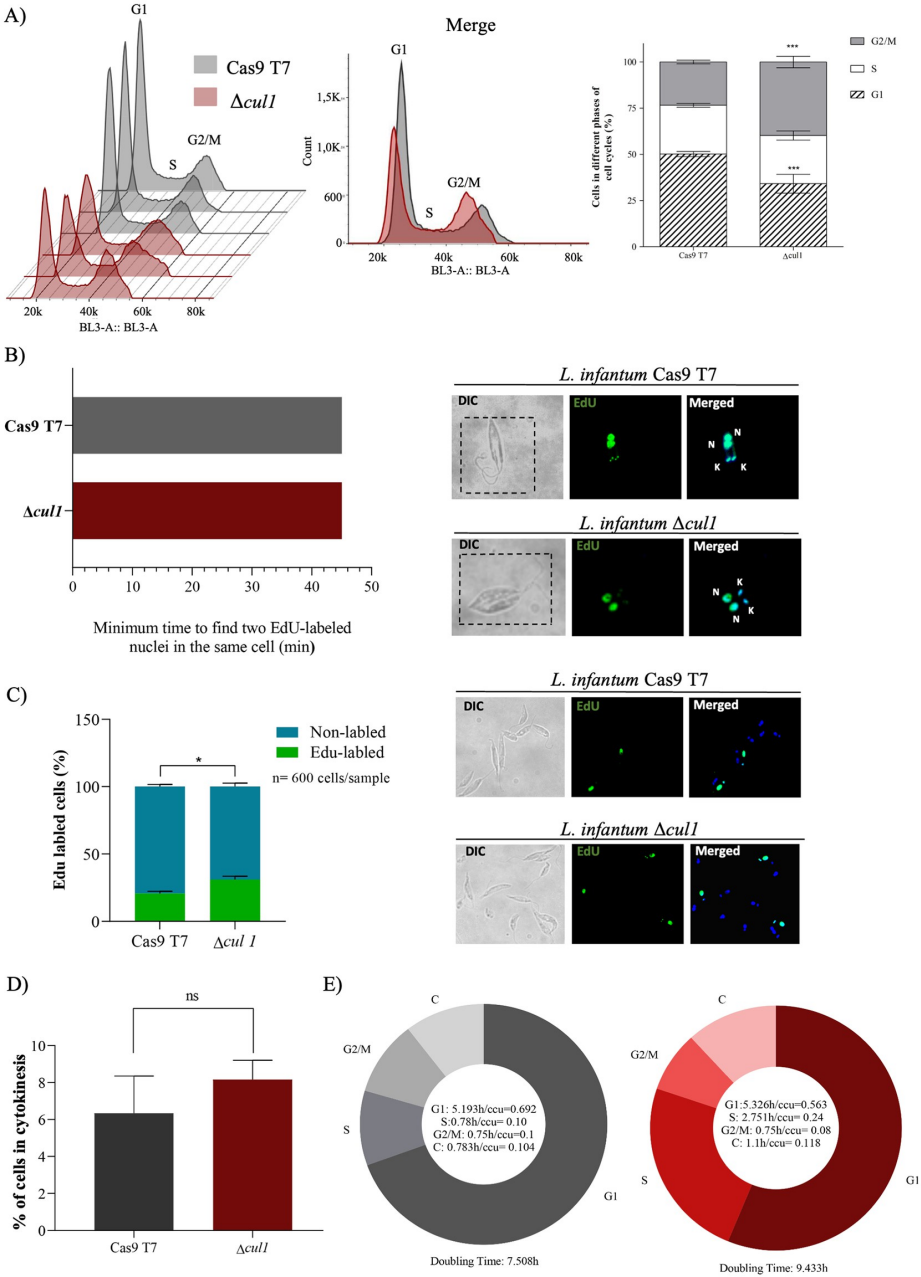
Cell cycle analysis of *L*. *infantum* Δc*ul1* and the parental cell line. A) Flow cytometry analysis and quantification of DNA content. B) Estimation of the duration of the G2+M phases through the minimum time to find two EdU-labeled nuclei in the same cell. C) Percentage of EdU-labeled cells after 1 h EdU pulse. D) DAPI-labeled parasites with 2K2N configurations were used to estimate the percentage of cells performing cytokinesis. E) The CeCyD software [47] was used to estimate the cell cycle phase duration. Representative images were obtained with 100X oil objective. EdU-labeled cells were revealed using azide conjugated with Alexafluor 488 (green). DAPI (blue) was used to stain DNA-containing organelles: kinetoplast (K) and nucleus (N). For A, three biological replicates were performed, and statistical significance was determined using the TWO-WAY ANOVA test (*p<0.05; ***p<0.001). For C and D, unpaired t-test was used (n = 3). Graphs were generated using GraphPad Prism v5.0 software.

DNA content analysis by flow cytometry revealed a similar profile between Δ*cul1* and the parental cell lineage (Fig 8A, left). However, the overlap among the histograms revealed an accumulation of the cells in late S and G2/M phases in *L*. *infantum* Δ*cul1* (Fig 8A, middle). Analysis of cell distribution showed a significant reduction in 2n content (equivalent to G1/G0 phase) and an increase in 4n content (equivalent to late S or G2/M/C phases) in the knockout cells (Fig 8A, right). These results suggest that LinfCul1 influences the cell cycle progression. To investigate the progression of the S phase and infer the duration of the cell cycle phases, we used 5-ethynyl-2’-deoxyuridine (EdU) a thymidine analog to monitor DNA replication. For both lineages, EdU-labeled 2K2N parasites were first detected 0.75 h (45 min) after EdU addition, indicating that cells at the end of S phase required 45 min to proceed through G2 and M phases (Fig 8B). Next, we measured the proportion of EdU-labeled parasites after 1 h EdU pulse. Interestingly, *L*. *infantum* Δ*cul1* showed slightly more Edu-labeled cells than the parental cell lineage (Fig 8C). Also, both lineages showed a similar proportion of cells in cytokinesis (2K2N) (Fig 8D). Based on these data, S-phase duration was estimated using CeCyD software [47]. We observed a significant increase in the duration of the S phase for the *L*. *infantum* Δ*cul1* lineage (S phase was estimated to be 2.751 h, or 0.24 ccu, for Δ*cul1* lineage, while the parental lineage presented an S phase equal to 0.78 h, or 0.1 ccu) (Fig 8E). Thus, our findings show that the increase in 4n content observed in Δ*cul1* lineage (Fig 8A) is actually cells accumulated in the late S phase, given that the end of this phase includes duplicated DNA content (4n), and its transition to G2 is not easily identified by flow cytometry. In other words, our results suggest that LinfCul1 regulates the S phase progression and possibly the transition between the late S to G2 phase in *L*. *infantum* through a mechanism that still needs to be investigated since there is no description of S/G2 checkpoint in trypanosomatids.

## Discussion

Cullin-RING ubiquitin ligases (CRLs) mediate ubiquitination of protein substrates to regulate many aspects of eukaryotic biology, including cell division, signal transduction, transcription, metabolism, hormone perception, circadian rhythms, differentiation, and development [24]. Many viruses and bacteria exploit CRLs activities to neutralize host antimicrobial defenses and facilitate their own replication [48]. In *Plasmodium berghei*, CRL1(FBXO1) and SKP1/Cullin1/FBXO1 (SCF^FBXO1^) complex are regulators of cell division and are essential for parasite-specific processes in the mammalian host and mosquito [49]. *T*. *brucei* SKP1-Cullin-F-box (SCF) regulates cell cycle progression, where TbSkp1 participates in the G1/S transition, TbRbx1 in kinetoplast DNA (kDNA) replication, and the E2 CDC34 ubiquitin-conjugating enzyme is related to cytokinesis [45]. As CRLs play crucial roles in a variety of organisms, we explored whether this class of E3 ubiquitin-ligases is conserved in *Leishmania* parasites. The orthologous genes of *H*. *sapiens SKP1*, *CUL1*, and *RBX1* were conserved and were found in the *Leishmania* genus. Additionally, the amino acid residues responsible for SKP1, CUL1, and RBX1 interactions to assemble the CRL complex were conserved. This evolutionary conservation was corroborated by phylogenetic analysis, in which inferred phylogenies for SKP1, CUL1, and RBX1 appropriately reconstructed the known evolutionary relationships among *Leishmania* species. Notably, the large CUL1 protein is highly conserved across species.

The predicted three-dimensional structures of LinfSkp1, LinfCul1, and LinfRbx1 generated by AlphaFold [35,36], compared to their orthologs in *H*. *sapiens*, revealed a high degree of structural similarity in the high-confidence index region. The RMSD values suggest that LinfSkp1 closely resembles *H*. *sapiens* SKP1, whereas LinfCul1 and LinfRbx1 exhibit minimal structural disparities, which is interesting for selective drug development targeting LinfCRL1. Strikingly, the *in silico* assembly of the LinfCRL1 complex superimposed with the human CRL1(SKP2) or SCF^Skp2^ crystal structure [17] demonstrated the same structural topology, with LinfCul1 working as a scaffold for the complex, binding to LinfSkp1 at its N-terminus and LinfRbx1at its C-terminus. To confirm these *in silico* data, co-IP assays were performed in HEK293T cells transfected with plasmids encoding the genes of interest. This mammalian cell line is highly transfectable and produce large amount of protein by culture being used as a platform to produce recombinant proteins [50]. Co-IP results demonstrated that the conservation of interaction regions of LinfCRL1 proteins compared to *H*. *sapiens* was sufficient for their interactions and assembly of the complex. In addition, they revealed interactions among the components of *L*. *infantum* and *H*. *sapiens*, which might be explored by the parasite to evade the host immune system. *Leishmania* uses different survival strategies to evade or modulate host immune defense by regulating the ubiquitin proteasome system, which is responsible for parasite autophagy, DNA repair, and protein trafficking [51]. *L*. *donovani* controls the deubiquitinating enzyme A20 to hinder TLR2-mediated proinflammatory gene expression, thereby evading host immune responses [52]. Hepcidin-mediated degradation of ferroportin (Fpn) in macrophages is a widely adopted strategy to restrict iron availability to invading pathogens. *L*. *donovani* modulates the level of an F-box protein (FBXL5) in the host macrophage to activate IRE-IRP interaction, leading to the inhibition of Fpn. This inhibition promotes evasion of *L*. *donovani* from the immune system [53]. Thus, our findings open perspectives for the exploration of host CRLs components by *Leishmania infantum* with consequences for the disease caused by the parasite.

To evaluate the assembly of the LinfCRL1 complex, the interactome of LinfSkp1 and LinfCul1 was acquired via immunoprecipitation followed by direct on-bead digestion and mass spectrometry protein identification. Remarkably, LinfCul1 and LinfRbx1 were identified in the LinfSkp1 interactome, and LinfRbx1 and LinfSkp1 were recovered from the LinfCul1 interactome confirming the presence of the LinfCRL complex in this parasite. Functional CRL1 complexes are conjugated to F-box proteins, which serve as specificity factors for this complex. These F-box proteins interact with SKP1 through the F-box domain and engage with their substrates through other domains [15–17]. Interestingly, five F-box proteins (LinfFlp1, LinfFlp2, LinfFlp3, LinfFlp5, and LinfFlp6) were directly identified in the LinfSkp1 interactome, whereas LinfFlp4 was indirectly identified in the LinfCul1 interactome. The interaction of LinfFlp1 to 6 with LinfSkp1 was validated through co-IP assays, confirming the assembly of the LinfCRL1 complex with F-box proteins. Strikingly, an *in vitro* ubiquitination assay using *in vitro* purified LinfCRL(Flp1) demonstrated its capability to catalyze ubiquitin transfer. Molecular dynamic simulation of LinfCRL1 with the predicted F-box motif of Flp1(Flp1^1–100^) demonstrated its interaction with LinfSkp1. However, whether this motif is sufficient for the interaction of Flp1 with LinfSkp1 needs to be studied. A bending movement of LinfCul1, bringing LinSkp1 and LinfRbx1 together, was also observed. Whether this conformational movement mediates the transfer of ubiquitin from E2 bound to LinfRbx1 to a substrate recruited by LinfSkp1 has to be explored. The human F-box proteins CRL(SKP2) (FBXL1) and CRL(β-TRCP) (beta-transducin repeat-containing protein) (FBXW1A) serve as primary regulators of the eukaryotes cell cycle [24]. Additionally, various other CRL-associated F-box proteins regulate a myriad of cellular processes in mammals [54], demonstrating their essential roles. This class of proteins remains unexplored in *Leishmania* spp. and only the F-box protein CFB2 has been studied in *Trypanosoma brucei* and described as required for cytokinesis of its bloodstream form [55]. Having shown their involvement in the assembly of LinfCRL1, investigation of their roles in these parasites is an intriguing area of investigation. We did not identify orthologs of *H*. *sapiens* F-box proteins in *Leishmania* spp., revealing poor sequence conservation in this class of proteins in these organisms, except for the expected conservation of the F-box domain. Consequently, we argue that LinfFlps are interesting targets for the development of selective anti-leishmanial drugs.

In the LinfSkp1 interactome, the presence of nucleic acid-binding proteins is noteworthy, with most of them being RNA binders, particularly the genes (LINF_260007900, LINF_320042200, LINF_330027700, LINF_320012500, LINF_330034300, and LINF_070016300). RNA-binding proteins (RBPs) in *Leishmania* spp. function as trans-regulators, overseeing the processing and trafficking of RNA molecules from synthesis to degradation. LINF_260007900 (Cleavage and Polyadenylation Specificity Factor Subunit 5) could potentially constitute a component of the cleavage factor Im (CFIm) complex, which acts as an activator in the pre-mRNA 3’-end cleavage and polyadenylation processes essential for the maturation of pre-mRNA into functional mRNAs. Five components of the Cleavage and Polyadenylation Specificity Factor complex (CPSF) were identified and confirmed through reciprocal co-immunoprecipitation in *L*. *mexicana*, which play a role in co-transcriptional cleavage and polyadenylation [56]. Interestingly, the interactome of non-poly(A) RNA in *L*. *mexicana* revealed the presence of Skp1 and Cul1 [57], suggesting that the CRL complex may be involved in transcriptional regulation in *Leishmania* spp. In addition, LINF_180009700 (Paralyzed flagella protein 20/Small ribosomal subunit protein RACK1/ Guanine nucleotide-binding protein subunit beta-like protein) could be part of the core ribosomal proteins that repress gene expression and is involved in the induction of the ribosome quality control (RQC) pathway [58,59], which degrades nascent peptide chains during problematic translation [60]. Intriguingly, the majority of the LinfCul1 interactome comprises ribosomal proteins, indicating a correlation between CRL1 with translational regulation and RQC in *Leishmania* spp.

The formation of *H*. *sapiens* CRL1 complexes is governed by the covalent attachment of a ubiquitin-like protein, namely NEDD8, at amino acid residue 720 (K720) in the C-terminus of Cullin1. Neddylation is a process akin to ubiquitination, involving the E1, E2, and E3 classes of enzymes. This process is hindered by the binding of CAND1 (cullin-associated and neddylation-dissociated 1) protein to Cullins, inhibiting complex assembly. The specific isopeptidase activity of the COP9 signalosome (CSN) complex facilitates the removal of NEDD8 from Cullins, thereby enabling the binding of SKP1-F-box proteins to the CRL complex [61]. The E1 enzyme responsible for neddylation (UBA3) and the NEDD8-transferring enzyme UBC12 have been identified in the *L*. *mexicana* proteome [19] with their respective orthologs, LINF_010012200-T1 and LINF_240023100-T1, found in *L*. *infantum*. Here, we identified in LinfSkp1 and LinfCul1 interactome the gene LINF_340026400 (ubiquitin-like protein), which has not been explored. The BLAST search of this gene in UniprotDB identified the NEDD8 protein in different species of *Fusarium* fungus or the Ubiquitin-NEDD8-like protein RUB2 in *Nicotiana tabacum*. We speculated that the ubiquitin-like protein identified in the LinfCul1 interactome may serve as a regulator of the *L*. *infantum* CRL complex, functioning similarly to NEDD8 in *H*. *sapiens*. As expected, this protein was also present in the LinfSkp1 interactome, as the assembly of CRL requires the conjugation of NEDD8 to CUL1. Consequently, LinfCul1-NEDD8 co-eluted with LinfSkp1 in its interactome. Interestingly, the K720 residue is also conserved in *Leishmania* spp. CUL1, supporting the hypothesis that CRL1 is regulated by neddylation in these parasites.

The knockout of *LinfSKP1* and *LinfRBX1* results in nonviable *L*. *infantum* strains, demonstrating that they have essential roles in this parasite. Knockout of LmxM.31.0450 or LmxM.36.5820, two orthologous genes of *LinfSKP1* and *LinfRBX1* in *L*. *mexicana*, respectively, led to nonviable and viable parasites (LeishGEM data browser: https://browse.leishgem.org/). These outcomes may stem from the varied functions of RBX1 across different *Leishmania* species or from potential functional compensation upon knockout in *L*. *mexicana*. In *T*. *brucei*, TbRBX interacts with five distinct Cullins: TbCul-A, TbCulC, TbCulD, TbCulE, and TbCulF. This finding suggests that TbRBX plays various roles associated with multiple Cullins, underscoring its essential function in this parasite [62]. Conversely, SKP1 deletion resulted in nonviable lineages in both species, emphasizing the crucial role in these parasites. *L*. *infantum* Δ*cul1* was viable, but the strain exhibited impaired cell proliferation and an increased doubling time, suggesting that LinfCul1 plays some role in regulating the cell cycle progression. Interestingly, according to DNA content analysis and the EdU incorporation assay (Fig 8), LinfCul1 is possibly involved in S phase progression and in regulating the transition from the late S to G2 phase in this parasite, although there are no known control mechanisms for the S/G2 transition in trypanosomatids to date. In *T*. *brucei*, no phenotypic changes were observed after the knockout of TbCUL1, suggesting that it might be functionally redundant with other Cullins [45]. Whether the cell cycle effect of LinfCul1 is related to its function as a LinfCRL1 component or a non-canonical function has to be determined, since other CRL1 components have atypical functions such as SKP1 and F-box proteins [63,64]. In *L*. *donovani*, the cell cycle is regulated by the Cdc20 protein (LDCdc20p), a regulator of the Anaphase Promoting Complex/Cyclosome (APC/C), which mediates ubiquitin-dependent proteasomal degradation of key cell cycle regulators in eukaryotes [65]. CRL1 and APC/C are structurally similar E3 ubiquitin ligases that jointly regulate the cell cycle. While APC/C is active from mid-mitosis (anaphase) to the end of G1 phase, the CRL1 complex is functionally active from late G1 to early M phase [24]. Interestingly, *L*. *infantum* exhibits nine orthologous genes in the *H*. *sapiens* APC/C complex (LINF_350050800, LINF_350042900, LINF_240023200, LINF_300024800, LINF_050009100, LINF_300012300, LINF_300035500, LINF_120011300, and LINF_040009000), implying that the cell cycle may be regulated by the CRL1 and APC/C complexes.

The *L*. *infantum* Δ*cul1* line showed parasite agglomeration as promastigotes, reminiscent of rosette formation. These structures have been described in *L*. *major* expressing polysialic acid (PSA) on the cell surface as well as acetylated neuraminic acid (NeuPSA) contained within PSA. The frequency of rosette formation and the expression of PSA/NeuPSA on the cell surface were temperature dependent. Rosettes form structures resembling cell fusion bodies that are then released. It is hypothesized that rosettes represent an unrecognized phase in the life cycle of *Leishmania* spp., starting the mating process, during which the expression of PSA/NeuPSA plays a crucial role in *L*. *major* [66]. In addition to temperature stress, rotational movement of the cell culture also influences the formation of cellular aggregates, similar to rosettes [67]. The structures observed in the Δ*cul1* strain resemble those observed in *L*. *major*, with intertwining flagella and fusion bodies. The initial observation of potential sexual reproduction in *Leishmania* was derived from quantitative microspectrophotometry, suggesting the occurrence of nuclear fusion or sexual reproduction within the intracellular amastigote form [68]. The identification of hybrid genotypes in natural isolates of *Leishmania* has further supported this hypothesis, and computational methods have been employed to investigate aneuploid genome dynamics [69]. Genetic exchange in *Leishmania* can be facilitated by IgM antibodies, which induce the formation of spherical parasite clumps, thereby promoting the fusion of parasites and formation of hybrids [70]. HOP1 and a HAP2 paralog (HAP2-2) have been shown to be essential components of the *Leishmania* meiosis machinery and the cell-to-cell fusion mechanism associated with sexual reproduction in these parasites [71]. Our results demonstrate that knockout of *LinfCUL1* stimulates the formation of rosettes, indicating that the expression of this gene represses or reduces the formation of these structures. The Cullin protein family has been reported to be associated with the regulation of meiosis and chromosome segregation in different organisms. Cullin9 protects mouse eggs from aneuploidy by controlling microtubule dynamics during oocyte meiosis [72], and Cullin 4A regulates meiotic progression in mouse spermatogenesis [73]. SCF/Skp1 together with Fbh1 ensures proper chromosome segregation in fission yeast [74]. In *Caenorhabditis elegans*, CUL-2 controls various crucial processes in cell division and embryonic development, including meiotic progression, anterior–posterior polarity, and mitotic chromatin condensation [75]. Whether *CUL1* or the CRL1 complex is responsible for the division of fused parasites during sexual reproduction of *Leishmania* remains to be explored.

In summary, we demonstrated that the *L*. *infantum* genes LINF_110018100 (SKP1-like protein), LINF_240029100 (cullin-like protein), and LINF_210005300 (putative ring-box protein 1) form a LinfCRL1 complex in combination with six F-box proteins (Flp1-6), potentially regulated by a NEDD8-like protein LINF_340026400. The LinfCRL1 complex, when conjugated to Flp1, exhibited activity in *in vitro* ubiquitination assays. LinfSkp1 and LinfCul1 interactomes revealed proteins associated with diverse cellular processes, suggesting their roles in transcription and translation, respectively. The knockout of *LinfSKP1* and *LinfRBX1* produced nonviable lineages, whereas *L*. *infantum* Δ*cul1* was viable, with an impaired proliferation rate, cell cycle phase alterations, and stimulation of rosette formation. Thus, we characterized Cullin1-RING ubiquitin ligases or SCF1 in *L*. *infantum*, as well as their protein partners. These findings not only advance our understanding of the molecular machinery governing *Leishmania* biology but also provide potential targets for therapeutic intervention against visceral leishmaniasis.

## Materials and methods

### Parasite cultures and proliferation

The *Leishmania infantum* isolate HU-UFS14, characterized by experimental infections [76–78] and whole-genome sequencing [79], was used. Promastigotes were cultivated and maintained weekly at 27°C in Schneider’s Drosophila Medium (Gibco) supplemented with 20% heat-inactivated fetal bovine serum, 1% penicillin/streptomycin/L-glutamine solution (Gibco), and 2% filtered male urine [80]. Promastigotes at a density of 1 × 10^5^ cells/mL were added to 12-well plates in triplicate, and an aliquot from each well was counted daily for 9 days to determine the growth.

### *L*. *infantum* growth and duplication rate

Promastigote cultures of wild-type *Leishmania infantum* HU-UFS14, *L*. *infantum* Cas9 T7 and *L*. *infantum* knockout for the gene LINF_240029100 (*L*. *infantum Δcul1*::*PUR/Δcul1*::*BLA*), or cell lines *L*. *infantum 3xmyc-mCherry*::*SKP1*, and *L*. *infantum 3xmyc-mCherry*::*CUL1* were used. These strains were plated in triplicates in 12-well plates containing 1 x10^5^ cells/mL in complete M199 medium (20% FBS and 1% penicillin-streptomycin). The cell lines were cultivated in the following antibiotics: *L*. *infantum Cas9 T7*, hygromycin (60 μg/ml); *L*. *infantum Δcul1*, hygromycin (60 μg/ml), puromycin (40 μg/ml) and blasticidin (60 μg/ml), and *L*. *infantum 3xmyc-mCherry*::*SKP1* and *L*. *infantum 3xmyc-mCherry*::*CUL1* with hygromycin (60 μg/ml) and puromycin (40 μg/ml). The promastigote plates were kept in incubators at 27 °C with 5% CO_2_, and cell counting was performed every 24 h for 8 days in a Neubauer chamber diluting samples 100x in PBS 1X with 2% formaldehyde. The plot and data analyses were performed using GraphPad Prism v5.0, using the two-way ANOVA statistical test.

Duplication rate (DR) or population doubling (PD) assays were carried out as [81], and 1 × 10^5^ cells/mL were cultivated in M199 supplemented with the antibiotics mentioned above. Each strain was cultivated in 12 wells plate and the cell number was determined daily, and the same number of cells were seeded per well for 8 consecutive days. PD was determined as Log_2_ [TCN/(2 × 10^5^) per day] = growth rate (GR) (TCN = Total Cell Number). Thus, the GR on day 2 (GRD2) was GRD1+GRD2, and GRD3 = GRD2+GRD3, among others. The values plotted on the graph are the means of triplicates for each time point for each day, and two-way ANOVA was used in GraphPad Prism v5.0.

### Generation of the CRISPR-Cas9 transgenic lines of *L*. *infantum* for CRL1 genes and selection

Using a CRISPR-Cas9-based approach [39], an *L*. *infantum* transgenic line expressing Cas9 and T7 RNA polymerase (Cas9 T7) was generated for N-terminal tagged *L*. *infantum* genes SKP1-like protein (LINF_110018100) and cullin-like protein (LINF_240029100), orthologs of the *H*. *sapiens SKP1* and *CUL1*, genes respectively. For generation of this transgenic line expressing Cas9 and T7 RNA polymerase, *L*. *infantum* HU-UFS14 promastigotes in logarithmic phase were transfected with 2 μg of pTB007 plasmid (containing these both genes) in 1x Tb-BSF buffer (90 mM sodium phosphate, 5 mM potassium chloride, 0.15 mM calcium chloride, 50 mM HEPES) [82] in 2 mm gap cuvettes with Gene Pulser Xcell Electroporation Systems (Biorad) using one pulse of 0,5 KV and 0,5 μF [83]. Transfected promastigotes were recovered and after 24 h, 60 μg/mL hygromycin B was added as the selection drug. This transgenic line was used for N-terminal tagged lines using the pPLOT plasmid for amplification of the linear cassette and pT plasmid (pTpuro and pTblast) was used for gene knockout. Primers sequences were obtained in http://www.leishgedit.net/Home.html [34] using the follow genes IDs: (LinJ.11.1200) (LINF_110018100) (SKP1-like protein), (LinJ.24.2380) (LINF_240029100) (cullin-like protein-like protein) and (LinJ.21.0040) (LINF_210005300) ring-box protein 1 –putative (S3 Table). For gene tagging, the repair cassettes were amplified by PCR, using 75 ng of circular pPLOTv1 puro-mCherry plasmid as a template and 10 μM gene-specific forward and reverse primers with Phusion Flash High-Fidelity Master Mix (Thermo Scientific-F548L). To amplify sgRNA templates, 10 μM of G00 (sgRNA scaffold) [34] and 10 μM of each 5’ or 3’ primer were used. All PCR amplicons were precipitated with ethanol before parasites transfection. To screen for CRISPR-Cas9 tagged or knockout genes in *L*. *infantum* cell lines, genomic DNA was isolated from parasites using the Genomic DNA Purification System (Promega) kit and used as a template for PCR reactions with *Taq* DNA polymerase (Thermo Scientific). Primer sequences are described in S4 Table. For tagging or gene knockout, approximately 1,2 ×10^7^ cells were transfected with 2 μg PCR amplicons for sgRNA, and donor DNA and transfectants were then selected with 40 μg/mL puromycin dihydrochloride and/or 60 μg/mL blasticidin S HCl.

### Mammalian cell culture and transfection

HEK293T (Human Embryonic Kidney 293T) cells were obtained from BCRJ (Banco de Células do Rio de Janeiro). The cells were cultured in a 5% CO_2_ atmosphere in DMEM high glucose (Corning) supplemented with 10% fetal bovine serum (FBS, Gibco), and penicillin (100 units/mL), streptomycin (100 μg/mL), and L-glutamine (0.292 mg/mL) (Thermo Fisher Scientific). To replicate cells, they were washed once with Phosphate Buffered Saline 1x (PBA) (HyClone) and detached with trypsin (TrypLe Express, Thermo Fisher Scientific). Plasmid constructs were transfected into 60–80% confluent cells using DNA-PEI (polyethylenimine) (PEI 1 μg/μL, pH 7.2) at a ratio of 1:3 in OptiMEM (Thermo Scientific) for 24 h.

### Co-immunoprecipitation of HEK293T transfected cells

To evaluate whether CRL1 components of *L*. *infantum* have conserved the interaction region among their proteins, co-immunoprecipitation assays in HEK293T cells transfected with plasmids encoding each gene were performed between parasite CRL1 complex component and the human ortholog partner protein. *L*. *infantum* genes were amplified by PCR from the genomic DNA of the HU-UFS14 isolate and cloned into pcDNA3.1(+) as follows: pcDNA3.1(+)-SKP1-like protein-HA (LinfSkp1-HA), pcDNA3.1(+)-SKP1-like protein-FLAG (LinfSkp1-FLAG), pcDNA3.1(+)-cullin-like protein-like protein -FLAG (LinfCul1-FLAG), pcDNA3.1(+)-cullin-like protein-like protein -myc (LinfCul1-myc) and pcDNA3.1(+)-ring-box protein 1 –putative-HA (LinfRbx1-HA) (primers are detailed in S5 Table). The *H*. *sapiens* genes *FBXO7*, *SKP1*, *CUL1*, and *RBX1* were previously described [34] and cloned into pcDNA3.1 to obtain the following plasmids: pcDNA3.1-2xFLAG-FBXO7, pcDNA3.1- CUL1-FLAG, pcDNA3.1-SKP1-HA and pcDNA3.1-RBX1-myc. For all co-immunoprecipitation assays cells were lysed with NP-40 lysis buffer (50 mM Tris-HCl pH 7.2, 225 mM KCl, and 1% NP-40) supplemented with the protease inhibitor cocktail *SIGMAFAST* (Sigma Aldrich) and phosphatase inhibitors (10 mM NaF and 1 mM Na_3_VO_4_) (Sigma Aldrich). Cell lysates were centrifuged 16,900 × g for 20 min at 4 °C, and approximately 3 mg of protein supernatant was subjected to immunoprecipitation (IP) with anti-FLAG agarose beads for 3 h at 4 °C. The eluates were obtained after elution with FLAG peptide (300 μg/mL) in FLAG elution buffer (HEPES 10 mM pH 7.9, MgCl_2_ 1.5 mM, KCl 225 mM and 0.1% NP-40) for 1 h at 4 °C. The eluates and inputs (100 μg or 3,3% of total cell lysates) were resolved using SDS-PAGE and subjected to western blotting with primary antibody incubation overnight at 4 °C. The following antibodies were used: anti-HA (#3724), anti-FLAG (#14793), anti-β-Actina (#3700), and anti-myc (#2272) from Cell Signaling Technology. Horseradish peroxidase (HPR)-conjugated anti-mouse (#0741806) or anti-rabbit (#0741516) both from KPL, were used as secondary antibodies for 1 h at room temperature. Western blotting images were captured using a ChemiDoc XRS+ (BioRad).

### Immunoprecipitation of LinfSkp1 and LinfCul1

Three independent replicates of *L*. *infatum 3x myc-mCherry*::*SKP1* or *L*. *infatum 3x myc-mCherry*::*CUL1* and two independent replicates of *L*. *infantum* Cas9 T7 as a negative control were used. For immunoprecipitation, 4 × 10^9^ parasites were washed twice in PBS and lysed with cold NP-40 lysis buffer (50 mM Tris-HCl pH 7.2, 225 mM KCl, and 1% NP-40) supplemented with a protease inhibitor cocktail *SIGMAFAST* (Sigma Aldrich) and phosphatase inhibitors (10 mM NaF and 1 mM Na_3_VO_4_) with three cycles of freezing in liquid nitrogen and thawing at room temperature. Later, the lysates were maintained on ice for 30 min and then centrifuged for 20 min at 14,000 × g at 4 °C. The supernatant protein concentration was determined using Bradford (Sigma Aldrich). For each immunoprecipitation, 20 mg of total protein was incubated with 60 μL of anti-myc beads (Sigma Aldrich, #E6654) for 3 h at 4 °C with rotation. Beads were washed with 5 × 500 μL ice-cold lysis buffer and 3 × 500 μL ice-cold wash buffer (10 mM HEPES pH 7.9, 1.5 mM MgCl_2_, 225 mM KCl) following on-bead digestion [40].

### Sample preparation for mass spectrometry analyzes

Trypsin digestion was performed by resuspending beads in 93 μL of 50 mM ammonium bicarbonate and 1 μL of 0.5 M dithiothreitol (DTT), and the samples were incubated at 56°C for 20 min. Next, 2.7 μL of fresh 0.55 M iodoacetamide was added, and the samples were incubated at room temperature for 15 min protected from light. That, 1,3 μL of trypsin gold, mass spectrometry grade (Promega V5280), was added, and the samples were mixed and incubated overnight at 37°C. The reaction was stopped by the addition of 1 mL of trifluoroacetic acid (liquid chromatography-mass spectrometry [LC-MS] grade), and incubated for 5 min [40]. Then, the samples were desalted using Stage Tips with C18 membranes (Octadecyl C18-bonded silica – 3M Empore extraction disks) and completely dried in an evaporator (SPD 1010 SpeedVac, Thermo).

### Analysis by Nanoflow nLC–MS/MS

The samples were reconstituted in 10 μL of formic acid, and an aliquot of 1 μL was analyzed using an ETD-enabled Orbitrap Velos mass spectrometer (Thermo Fisher Scientific, Waltham, MA, USA) coupled to an EASY-nLC system (Proxeon Biosystem, West Palm Beach, FL, USA) via a Proxeon nanoelectrospray ion source. Peptides were separated using an analytical column, PicoFrit Column (20 cm × ID75 μm, 5 μm particle size, New Objective), employing a 2–90% acetonitrile gradient in 0.1% formic acid at a flow rate of 300 nL·min−1 over a 65-minute duration. The nanoelectrospray voltage was set to 2.2 kV, and the source temperature was maintained at 275 °C. The instrument methods were configured in a data-dependent acquisition mode. Full-scan MS spectra (m/z 300–1600) were acquired using the Orbitrap analyzer after accumulation to a target value of 1 × 10^6^. The resolution in the Orbitrap was set to r = 60,000, and the 20 most intense peptide ions with charge states ≥2 were sequentially isolated to a target value of 5,000 and fragmented in the linear ion trap using low-energy CID (normalized collision energy of 35%). The signal threshold for triggering an MS/MS event was established at 1,000 counts. Dynamic exclusion was activated with an exclusion size list of 500, an exclusion duration of 60 s, and a repeat count of 1. An activation of q = 0.25 and activation time of 10 ms were applied [84].

### Raw LC–MS/MS data analysis

Protein identification was conducted using Proteome Discoverer version 1.4 (Thermo Fisher Scientific), employing the Sequest search algorithm. The analysis was performed using the *Leishmania infantum* protein database (release 2022; 8272 sequences; 5263968 residues) obtained from UniprotDB. Carbamidomethylation was designated as a fixed modification, whereas methionine oxidation was considered a variable modification. Parameters for protein identification included a maximum of two trypsin missed cleavages and tolerance levels of 10 ppm for precursor mass and 1 Da for fragment ions. A filtering criterion was applied to maintain a maximum false discvery rate of 1% at both peptide and protein levels. Proteins identified in at least one negative control were excluded from those identified in the SKP1 and Cul1 interactomes. Proteins identified with at least one unique peptide present in all three samples of each interactome in triplicate were considered hits and are listed in S2 Table.

### Phylogenetic analysis

Nucleotide sequences of each human gene were used to search for orthologs in 12 *Leishmania* species (*L*. *braziliensis*, *L*. *panamensis*, *L*. *amazonensis*, *L*. *mexicana*, *L*. *donovani*, *L*. *infantum*, *L*. *aethiopica*, *L*. *tropica*, *L*. *major*, *L*. *gerbilli*, *L*. *arabica*, and *L*. *turanica*) using blastx in TriTrypDB [85] (Target Data Type: Proteins; BLAST Program: blastx; Target Organism: *Leishmania*). The sequences of SKP1, CUL1, and RBX1 human proteins and their orthologs in the 12 *Leishmania* species are listed in S1 Table. They were subjected to multiple sequence alignment (MSA), and the most appropriate amino acid substitution model was determined for each gene. CUL1 and RBX1 used the JTT matrix [30] and SKP1 used Le Gascuel [29] as an evolutionary model of amino acid substitution. For RBX1, a discrete gamma distribution was used to model evolutionary rate differences among sites (five categories (+G, parameter = 0.0575)). Phylogenetic reconstruction of each gene was performed with Maximum Likelihood (ML) method [86] using MEGA (version 11) and Bayesian Inference (BI) method using BEAST (version 1.10.4) [33]. For ML method the reliability of the obtained phylogeny was subsequently evaluated using the statistical bootstrap method [87], using a minimum of 1000 replicates. The bootstrap method generates data replicates from which multiple phylogenetic trees can be constructed. The bootstrap value associated with each branch of the tree reflects, as a percentage, the frequency with which a specific clade was observed in the replicates, indicating higher reliability in the phylogenetic relationships for higher values. For BI method, starting tree was random and molecular clock was strict as default [88]. Tree prior distribution was set to Yule speciation [89]. For each gene was performed 10^7^ iterations and sampling every 10^4^ iterations. For burn-in was set 10% of the iterations. Maximum Clade Credibility (MCC) trees were generated using TreeAnnotator using median heights for each gene. BI-inferred trees were visualized in FigTree (available at http://tree.bio.ed.ac.uk/software/figtree/).

### Protein structure modeling

The structures of the proteins encoded by genes the LINF_110018100, LINF_240029100, and LINF_210005300 from *Leishmania infantum* (JPCM5) were obtained from AlphaFold database [35,36] and subsequently compared to human orthologs of the CRL1 complex [17]. Amino acid residues were colored according to the reliability of their structure: dark blue for high reliability, light blue for moderate reliability, yellow for low reliability, and orange for very low reliability. The prediction of the CRL1-type E3 ubiquitin ligase complex was performed using the AlphaFold2 (AF2) (AlphaFold v2.3.2 online version) [35]. The obtained models were visualized and analyzed using UCSF Chimera software (version 1.17.3) [90]. RMSD was calculated using the DALI server, a server for comparing protein structures in 3D [91]. For identical structures, the RMSD is 0, and values between 0 and 2 Å indicated a high percentage of identity [37].

### Molecular Dynamics (MD) simulation

The AF2 implemented in ColabFold [92] was used for generation of LinfCRL1 complex structure including the F-box motif of Flp1(1–100). This model were used for the MD simulations using the AMBER 22 package [93–95]. The protonation states of the residues were defined using PDB2PQR [96]. The AMBER FF19SB force field was employed along with the OPC water model. The system was immersed in a cubic box filled with water, Na^+^, and Cl^−^ to neutralize the system and maintain a salt concentration of 150 mM. The system energy was minimized in 8,000 steps of energy minimization, followed by heating to 300 K in 100 ps of the NVT simulation. The system density was then equilibrated by 100 ps of NPT simulation, followed by a 2 ns final equilibration in the NPT ensemble. A 250 ns simulation of the equilibrated system in the NPT ensemble was used for analysis. The entire system containing approximately 850,000 atoms was simulated using the hydrogen mass repartition scheme [97] and a 4 fs timestep. Analysis of the obtained trajectory file was performed using CPPTRAJ [98] and AmberEnergy++ [99].

### *In vitro* ubiquitination assay

HEK293T cells were transfected with LinfCRL1 (Flp1) and rinsed with the lysis buffer. The supernatants were immunoprecipitated using anti-FLAG M2 beads, and the complex was eluted with 100 μg/ml of 3XFLAG peptide (Sigma-Aldrich) in Tris-buffered saline (TBS; 50mM Tris-HCl, pH 7.5, 0.15 M NaCl). The *in vitro* ubiquitination reactions were developed by 90 min at 30 °C incubation of purified LinfCRL1 (Flp1) with ubiquitin mix containing by reaction 100 nM of E1 (#E-304), 500 nM of E2 (UbcH5c/UBE2D3)(# E2-627), 20 μM of ubiquitin (#U-100H) and 1X ubiquitination buffer [34]. Where indicated, ATP was used at 2mM (#B-20). The UBE2D3 is an ortholog of UBC13 in *L*. *infantum* (LINF_350017900). All ubiquitination reagents are from *H*. *sapiens*, and they were purchased from Boston Biochem. The proteins were loaded on SDS-PAGE, and the western blots were probed with anti-ubiquitin antibodies (Cell Signaling Technology).

### Rosette formation analysis

The cell lines were seeded in triplicate in 25 cm^2^ culture flasks containing 2 x10^5^ cells/mL in 5 mL of complete M199 supplemented with 0,1 mM adenine, 2,5 mg/ml hemin, 1% penicillin-streptomycin, and 20% FBS, with the respective selection antibiotics added. Triplicate flasks for each cell line were observed, photographed, and filmed per field (four corners of the flask and its central portion, totaling five fields) every 24 h for 7 days, using OPTIKA PROVIEW v4.11 software via the C–B5 OPTIKA Microscopes ITALY camera, with a magnification of 20X. Data were obtained from the analysis of five regions, including the four corners and central portion of the culture flask. The rosette count was normalized to the number of photographed fields per culture flask, and the values were plotted. Statistical differences and graph generation were assessed by two-way ANOVA variance using GraphPad Prism v5.0. To analyze the structure of rosette formation at higher magnification, a slide smear was prepared using the *L*. *infantum* Δ*cul1* strain. The samples were stained using the Romanowsky technique (Laborclin) and left to dry for 24 h. After this period, the slides were analyzed using an Olympus BX50 optical microscope with a 20X or 100X objective, and images were generated by the DP2-BSW v.2.2 software using the Olympus DP72 camera software.

### Flow cytometry

Promastigotes of *L*. *infantum* Cas9 T7 and *L*. *infantum Δcul1* were cultivated at a concentration of 2 x 10^7^ cells/ml. On the 2^nd^ day of the growth curve, the cells were harvested by centrifugation (1000 x g at 4 °C for 5 min) and washed in PBS at 4 °C. Subsequently, the cells were resuspended in 70% methanol (diluted in PBS) and incubated overnight at 4 °C. After this period, the cells were washed twice, and the pellet was resuspended in PBS containing propidium iodide (PI) (10 μg/ml) and RNase A (10 μg/ml) and incubated for 30 min at 37 °C. The parasites were analyzed using a flow cytometer (Attune Acoustic Focusing Flow Cytometer–Thermo Scientific). For each sample, 50,000 events were analyzed. The variance between these groups was similar. Images were drawn up using FlowJo v.7.6.5 software (FlowJo, LLC, Ashland, OR).

### Cell cycle analysis by EdU incorporation

Formaldehyde-fixed and DAPI-stained exponentially growing promastigotes were examined under a fluorescent microscope (Axiovert 200M –Zeiss), (100X oil objective) to observe the profile of organelles that contain DNA (nucleus and kinetoplast). The profile 2K2N was used to estimate the percentage of cells undergoing cytokinesis (C). Using this parameter for the samples analyzed, we estimated the duration of cytokinesis according to the Williams Equation [100]:

$$x=\frac{ln(1-y/2)}{-\alpha },$$

where x is the cumulative time within the cycle until the end of the stage in question, y is the cumulative % of cells up to and including the stage in question (expressed as a fraction of one unit), and α is the specific growth rate. To estimate the G2+M phase length, we added EdU to the medium containing exponentially growing promastigotes and collected samples every 15 min, proceeding with ‘click’ chemistry reaction, until a parasite containing two EdU-labeled nuclei (2K2N) was observed (corresponding to the length of G2+M phases). To determine the S-phase duration, we measured the proportion of EdU-labeled cells after 1 h EdU pulse. The S-phase duration was estimated according to the Stanners and Till Equation (Stanners and Till 1960) [101]:

$$S=\frac{1}{\alpha }ln[L+e^{\alpha (Z)}]-(Z+t),$$

where *L* is the proportion of cells exhibiting EdU-labeled nuclei, α = ln 2.T^-1^ (*T* = doubling time, expressed in h), Z = G2 + M + cytokinesis, and *t* is the duration of the EdU pulse in hours (h). Finally, the duration of the G1 phase was estimated by the difference between the doubling time (dt) and the sum of the other phases (S+G2+M+C). Of note, all these calculations were made with the help of the online software CeCyD, available at the address https://cecyd.vital.butantan.gov.br/ [47].

## Supporting information

S1 Table List of accession numbers of *SKP1*, *CUL1* and *RBX1* genes of *Leishmania* spp. used for phylogenetic analysis. (DOCX)

S2 Table *L*. *infantum* proteins identified in LinfSkp1 and LinfCul1 interactome. (DOCX)

S3 Table Primers used for tagging 3xmyc-mCherry into the N-terminus of *LinfSKP1* and *LinfCUL1* and generation of knockout lines for *SKP1*, *CUL1*, *and RBX1*. For N-terminal tagging, UFP (upstream forward primer), URP (upstream reverse primer), and 5 ‘sgRNA gene-specific primers were used. For knockout, UFP, DRP (downstream reverse primer), and both 5 ‘ and 3 ‘sgRNA gene-specific primers were used. For knockout, UFP, DRP (downstream reverse primer), and both 5 ‘ and 3 ‘sgRNA gene-specific primers were used. “Upstream” and “Downstream” primers contain primer binding site compatible with pT or pPLOT plasmids (lower case letters), as well as 30 nt homology arms for recombination (uppercase letters). “sgRNA” primers consisted of a T7 RNA polymerase target sequence, 20 nt overlap with the CRISPR-Cas9 backbone sequence (lower case letters), and a 20 nt sgRNA target sequence (uppercase letters).
(DOCX)

S4 Table Primers used to evaluate *L*. *infantum* Cas9T7 *3x myc-mCherry*::*SKP1* and *L*. *infantum* Cas9 T7 *3x myc-mCherry*::*CUL1* by PCR. (DOCX)

S5 Table Primers used for molecular cloning of *LinfSKP1*, *LinfCUL1* and *LinfRBX1* genes into pcDNA3.1(+)-HA/Myc/FLAG. (DOCX)

S1 Fig Phylogenies for CUL1 (A) and RBX1 (B) were inferred with a second method based on Bayesian Inference (BI) using BEAST program. Numbers next to the branches indicated the Bayesian probability values.
(TIFF)

S2 Fig The predicted three-dimensional structures of human SKP1, CUL1, and RBX1 were compared with those of *L*. *infantum* orthologs. A) *H*. *sapiens* SKP1 (P63208) and *L*. *infantum* (LINF_110018100). B) *H*. *sapiens* CUL1 (Q13616) and *L*. *infantum* (LINF_240029100). C) RBX1 (P62877) and *L*. *infantum* (LINF_210005300). Three-dimensional structures were generated using Alphafold [36, 50]. The reliability scale is depicted below according to the colors represented in the structures. RMSD values indicate the similarity between the structures.
(TIFF)

S3 Fig Prediction of LinfCRL1 complex using AF2. LinfSkp1, LinfCul and LinfRbx1 was used for the prediction of LinfCRL1. The model is colored by pLDDT on a red-to-blue scale, indicating an increase in the model reliability.
(TIFF)

S4 Fig A) Diagnostic PCR for confirmation of *L*. *infantum* Cas9 T7 *3x myc-mCherry*::*CUL1* and *3x myc-mCherry*::*SKP1* transgenic lines by amplification of the inserted donor DNA in the N-terminal region of *SKP1* and CUL1. B) Growth curves of *L*. *infantum* Cas9 T7 *3x myc-mCherry*::*CUL1* and *3x myc-mCherry*::*SKP1* compared to the *L*. *infantum* and *L*. *infantum* Cas9 T7 strains. C) Construction of the *L*. *infantum* Cas9 T7 *3x myc-mCherry*::*CUL1*::*HA-SKP1* lineage. D) Immunoblotting of the interaction between LinfFlp4 and LinfCul1 in HEK293T cells. HEK293T cells were transfected with the indicated plasmids (+), and cell lysates were immunoprecipitated with anti-FLAG beads and probed with the indicated antibodies.
(TIFF)

S1 Video Molecular dynamic simulation of LinfCRL1(Flp1^1–100^). (MP4)
