# Supplementary material for: Functional characterization of Cullin-1-RING ubiquitin ligase (CRL1) complex in Leishmania infantum
Source: PLoS Pathog. 2024 Jul 17;20(7):e1012336. doi: 10.1371/journal.ppat.1012336 (PMC11285970; doi:10.1371/journal.ppat.1012336)
Supplement: S1 Table — (DOCX) [file ppat.1012336.s001.docx]

**S1 Table 1**

|  | **SKP1** | **CUL1** | **RBX1** |
| --- | --- | --- | --- |
| *L. aethiopica* | LAEL147_000159400 | LAEL147_000398800 | LAEL147_000311800 |
| *L. amazonensis* | LAMA_000182000 | LAMA_000477400 | LAMA_000394800 |
| *L. arabica* | LARLEM1108_110018300 | LARLEM1108_240029700 | LARLEM1108_210005100 |
| *L. braziliensis* | LbrM.11.0950 | LbrM.24.2370 | LTULEM423_240030000 |
| *L. donovani* | LdBPK_111200.1 | LdBPK_242380.1 | LdBPK_210040.1 |
| *L. gerbilli* | LGELEM452_110018100 | LGELEM452_240030900 | LGELEM452_210005200 |
| *L. infantum* | LINF_110018100 | LINF_240029100 | LINF_210005300 |
| *L. major* | LmjF.11.1210 | LmjF.24.2290 | LmjF.21.0023 |
| *L. mexicana* | LmxM.11.1210 | LmxM.24.2290 | LmxM.21.0023 |
| *L. panamensis* | LPAL13_110015600 | LPAL13_240029900 | LPMP_210030 |
| *L. tropica* | LTRL590_110019300 | LTRL590_240030500 | LTRL590_210005400 |
| *L. turanica* | LTULEM423_110018400 | LTULEM423_240030000 | LTULEM423_210005200 |
