# Supplementary material for: Functional characterization of Cullin-1-RING ubiquitin ligase (CRL1) complex in Leishmania infantum
Source: PLoS Pathog. 2024 Jul 17;20(7):e1012336. doi: 10.1371/journal.ppat.1012336 (PMC11285970; doi:10.1371/journal.ppat.1012336)
Supplement: S2 Table — (DOCX) [file ppat.1012336.s002.docx]

**S2 Table 1**

| **Real targets LinfSkp1** | | | | |
| --- | --- | --- | --- | --- |
| **Uniprot** | **Gene ID** | **Name** | **Number of peptides** | **Functional classification** |
| [A0A6L0XQK8](https://www.uniprot.org/uniprotkb/A0A6L0XQK8/entry) | LINF_240029100 | Cullin-like protein-like protein | 23 | Ubiquitin proteasome system |
| [A0A6L0WUC5](https://www.uniprot.org/uniprotkb/A0A6L0WUC5/entry) | LINF_150014400 | Hypothetical protein - conserved | 15 | F-box protein/Ubiquitin proteasome system |
| [A0A6L0XEF5](https://www.uniprot.org/uniprotkb/A0A6L0XEF5/entry) | LINF_240015400 | F-box domain/F-box-like/Kelch motif/Galactose oxidase - central domain containing protein - putative | 15 | F-box protein/Ubiquitin proteasome system |
| [A0A6L0XWU9](https://www.uniprot.org/uniprotkb/A0A6L0XWU9/entry) | LINF_320019600 | Hypothetical protein - conserved | 14 | Uncharacterized |
| [A0A6L0XUF3](https://www.uniprot.org/uniprotkb/A0A6L0XUF3/entry) | LINF_340043300 | Phosphatidylinositol 4-kinase - putative | 14 | Protein kinase |
| [A0A6L0XNP9](https://www.uniprot.org/uniprotkb/A0A6L0XNP9/entry) | LINF_320042200 | RNA-binding - protein - putative | 13 | Nucleic acid binding |
| [A0A6L0XGJ7](https://www.uniprot.org/uniprotkb/A0A6L0XGJ7/entry) | LINF_230008800 | Hypothetical protein - conserved | 13 | Uncharacterized |
| [A0A6L0XCM5](https://www.uniprot.org/uniprotkb/A0A6L0XCM5/entry) | LINF_200008500 | Hypothetical protein - conserved | 12 | Uncharacterized |
| [A0A6L0WLY3](https://www.uniprot.org/uniprotkb/A0A6L0WLY3/entry) | LINF_050011500 | Ankyrin repeats (many copies)/Ankyrin repeats (3 copies)/Ankyrin repeat - putative | 11 | Protein binding |
| [A0A6L0XQ77](https://www.uniprot.org/uniprotkb/A0A6L0XQ77/entry) | LINF_330027700 | Ribosome assembly protein RRB1 – putative/ Histone-binding protein RBBP4 N-terminal domain-containing protein | 10 | Nucleic acid binding |
| [A0A6L0XHL7](https://www.uniprot.org/uniprotkb/A0A6L0XHL7/entry) | LINF_270019900 | Cupin-like domain/JmjC domain - hydroxylase - putative | 10 | Nucleic acid binding |
| [A0A6L0XRY9](https://www.uniprot.org/uniprotkb/A0A6L0XRY9/entry) | LINF_260007900 | Nucleotide hydrolase – putative/ Cleavage and polyadenylation specificity factor subunit 5 | 10 | Nucleic acid binding |
| [A0A6L0XH01](https://www.uniprot.org/uniprotkb/A0A6L0XH01/entry) | LINF_260030900 | Protein kinase - putative | 10 | Protein kinase |
| [A0A6L0WVU1](https://www.uniprot.org/uniprotkb/A0A6L0WVU1/entry) | LINF_140020700 | Hypothetical protein - conserved | 9 | Uncharacterized |
| [A0A6L0XFI3](https://www.uniprot.org/uniprotkb/A0A6L0XFI3/entry) | LINF_250026200 | Hypothetical protein - conserved | 8 | Nucleic acid binding |
| A0A6L0Y228 | LINF_360036800 | Hypothetical protein - conserved | 8 | Cilia and flagella |
| [A0A6L0XWM7](https://www.uniprot.org/uniprotkb/A0A6L0XWM7/entry) | LINF_320012500 | Mut7-C RNAse domain containing protein - putative | 7 | Nucleic acid binding |
| [A0A6L0Y1Z0](https://www.uniprot.org/uniprotkb/A0A6L0Y1Z0/entry) | LINF_360032300 | Hypothetical protein - conserved | 7 | F-box protein/Ubiquitin proteasome system |
| [A0A6L0WRD5](https://www.uniprot.org/uniprotkb/A0A6L0WRD5/entry) | LINF_140018100 | Ras-like small GTPases - putative | 7 | F-box protein/Ubiquitin proteasome system |
| [A0A6L0XLB3](https://www.uniprot.org/uniprotkb/A0A6L0XLB3/entry) | LINF_290033000 | MIZ/SP-RING zinc finger containing protein - putative | 6 | Nucleic acid binding |
| [A0A6L0XVN2](https://www.uniprot.org/uniprotkb/A0A6L0XVN2/entry) | LINF_310012600 | Hypothetical protein - conserved \| | 6 | Uncharacterized |
| [A0A6L0XC14](https://www.uniprot.org/uniprotkb/A0A6L0XC14/entry) | LINF_190021200 | Protein kinase - putative | 5 | Protein kinase |
| [A0A6L0XNV2](https://www.uniprot.org/uniprotkb/A0A6L0XNV2/entry) | LINF_340019100 | Hypothetical protein - [16–18] conserved | 5 | F-box protein/Ubiquitin proteasome system |
| [A0A6L0X5P6](https://www.uniprot.org/uniprotkb/A0A6L0X5P6/entry) | LINF_180009700 | Paralyzed flagella protein 20/Small ribosomal subunit protein RACK1 | 5 | Cilia and flagella |
| [A0A6L0XE26](https://www.uniprot.org/uniprotkb/A0A6L0XE26/entry) | LINF_230013300 | MYCBP-associated protein family - putative | 5 | Protein binding |
| [A0A6L0XN28](https://www.uniprot.org/uniprotkb/A0A6L0XN28/entry) | LINF_330034300 | Mitochondrial RNA binding complex 1 subunit | 4 | Nucleic acid binding |
| [A0A6L0Y088](https://www.uniprot.org/uniprotkb/A0A6L0Y088/entry) | LINF_340049700 | Protein serine/threonine phosphatase - putative | 4 | Protein phosphatase |
| [A0A6L0WQR2](https://www.uniprot.org/uniprotkb/A0A6L0WQR2/entry) | LINF_070016300 | RNA binding protein-like protein \| transcript_product | 4 | nucleic acid binding |
| [A0A6L0XW76](https://www.uniprot.org/uniprotkb/A0A6L0XW76/entry) | LINF_300031800 | Hypothetical protein - conserved | 3 | Protein binding |
| [A0A6L0XQB1](https://www.uniprot.org/uniprotkb/A0A6L0XQB1/entry) | LINF_340026400 | Ubiquitin-like protein | 3 | Ubiquitin proteasome system |
| [A0A6L0XUJ9](https://www.uniprot.org/uniprotkb/A0A6L0XUJ9/entry) | LINF_280036700 | Hypothetical protein - conserved | 3 | Uncharacterized |
| [A0A6L0XN75](https://www.uniprot.org/uniprotkb/A0A6L0XN75/entry) | LINF_330031500 | Hypothetical protein - conserved | 3 | Uncharacterized |
| [A0A6L0XNU8](https://www.uniprot.org/uniprotkb/A0A6L0XNU8/entry) | LINF_330005100 | Hypothetical protein | 3 | Protein binding |
| [A0A6L0XTV0](https://www.uniprot.org/uniprotkb/A0A6L0XTV0/entry) | LINF_360041200 | Enoyl-CoA hydratase/isomerase family/2-enoyl-CoA Hydratase C-terminal region containing protein - putative | 2 | metabolism |
| A0A6L0XFA3 | LINF_220021700 | Hypothetical protein - conserved | 2 | Uncharacterized |
| [A0A6L0XUD8](https://www.uniprot.org/uniprotkb/A0A6L0XUD8/entry) | LINF_300011600 | hypothetical protein - conserved | 2 | Uncharacterized |
| [A0A6L0XI75](https://www.uniprot.org/uniprotkb/A0A6L0XI75/entry) | LINF_270023100 | Eukaryotic translation initiation factor eIF-4E - putative | 1 | Nucleic acid binding |
| A0A6L0XS14 | LINF_350007000 | NLI interacting factor-like phosphatase - putative | 1 | Protein phosphatase |
| [A0A6L0XSA9](https://www.uniprot.org/uniprotkb/A0A6L0XSA9/entry) | LINF_360037100 | Hypothetical protein - conserved | 1 | Uncharacterized |
| [A0A6L0XK22](https://www.uniprot.org/uniprotkb/A0A6L0XK22/entry) | LINF_300037800 | Hypothetical protein - conserved | 1 | Uncharacterized |
| [A0A6L0XM95](https://www.uniprot.org/uniprotkb/A0A6L0XM95/entry) | LINF_300033400 | Hypothetical protein - conserved | 1 | Uncharacterized |
| [A0A6L0WK47](https://www.uniprot.org/uniprotkb/A0A6L0WK47/entry) | LINF_090013800 | Hypothetical protein - conserved | 1 | Cilia and flagella |
| [A0A6L0XC55](https://www.uniprot.org/uniprotkb/A0A6L0XC55/entry) | LINF_210005300 | Ring-box protein 1 - putative | 1 | Ubiquitin proteasome system |
| **Real targets LinfCul1** | | | | |
| [A0A6L0WUC5](https://www.uniprot.org/uniprotkb/A0A6L0WUC5/entry) | LINF_150014400 | hypothetical protein - conserved | 15 | F-box protein/Ubiquitin proteasome system |
| [A0A6L0WK79](https://www.uniprot.org/uniprotkb/A0A6L0WK79/entry) | LINF_110018100 | SKP1-like protein | 3 | Ribosomal proteins |
| [A0A6L0XN30](https://www.uniprot.org/uniprotkb/A0A6L0XN30/entry) | LINF_040009700 | 60S ribosomal protein L11 (L5 - L16) | 3 | Ribosomal proteins |
| [A0A6L0XXN9](https://www.uniprot.org/uniprotkb/A0A6L0XXN9/entry) | LINF_320009600 | 40S ribosomal protein S2 | 3 | Ubiquitin proteasome system |
| [A0A6L0XIZ9](https://www.uniprot.org/uniprotkb/A0A6L0XIZ9/entry) | LINF_290028700 | Domain of unknown function (DUF4139) - putative | 3 | Uncharacterized |
| [A0A6L0XA50](https://www.uniprot.org/uniprotkb/A0A6L0XA50/entry) | LINF_180019400 | 60S ribosomal protein L34 - putative | 2 | Ribosomal proteins |
| [A0A6L0XQB1](https://www.uniprot.org/uniprotkb/A0A6L0XQB1/entry) | LINF_340026400 | ubiquitin-like protein | 2 | Ubiquitin proteasome system |
| [A0A6L0XR67](https://www.uniprot.org/uniprotkb/A0A6L0XR67/entry) | LINF_350005600 | 2-oxoisovalerate dehydrogenase beta subunit - mitochondrial precursor - putative | 2 | Metabolism |
| [A0A6L0XN89](https://www.uniprot.org/uniprotkb/A0A6L0XN89/entry) | LINF_210018100 | 60S ribosomal protein L9 - putative | 2 | Ribosomal proteins |
| A0A6L0XQQ5 | LINF_250010600 | Scd6-like Sm domain containing protein - putative | 2 | RNA processing |
| [A0A6L0WUE6](https://www.uniprot.org/uniprotkb/A0A6L0WUE6/entry) | LINF_110017300 | 60S ribosomal protein L28 - putative | 2 | Ribosomal proteins |
| [A0A6L0XXY7](https://www.uniprot.org/uniprotkb/A0A6L0XXY7/entry) | LINF_330019800 | hypothetical protein - conserved | 2 | F-box protein/Ubiquitin proteasome system |
| [A0A6L0XYI1](https://www.uniprot.org/uniprotkb/A0A6L0XYI1/entry) | LINF_330042200 | beta prime cop protein - putative | 2 | Intracellular transport |
| [A0A6L0XGR3](https://www.uniprot.org/uniprotkb/A0A6L0XGR3/entry) | LINF_260024900 | hypothetical protein | 2 | Uncharacterized |
| [A0A6L0XSB9](https://www.uniprot.org/uniprotkb/A0A6L0XSB9/entry) | LINF_360039500 | cyclophilin 10 – putative/ Peptidyl-prolyl cis-trans isomerase | 1 | Protein folding |
| [A0A6L0XUB1](https://www.uniprot.org/uniprotkb/A0A6L0XUB1/entry) | LINF_360068000 | mkiaa0324 protein-like protein | 1 | Uncharacterized |
| [A0A6L0XC55](https://www.uniprot.org/uniprotkb/A0A6L0XC55/entry) | LINF_210005300 | Ring-box protein 1 - putative | 1 | Uncharacterized |
| [A0A6L0XTW0](https://www.uniprot.org/uniprotkb/A0A6L0XTW0/entry) | LINF_360052900 | 19S proteasome regulatory subunit | 1 | Ubiquitin proteasome system |
| [A0A6L0XNQ9](https://www.uniprot.org/uniprotkb/A0A6L0XNQ9/entry) | LINF_290016200 | hypothetical protein - conserved | 1 | Uncharacterized |
| [A0A6L0XF11](https://www.uniprot.org/uniprotkb/A0A6L0XF11/entry) | LINF_240005200 | Ribosomal protein L22p/L17e - putative | 1 | Ribosomal proteins |
| A0A6L0X3U7 | LINF_150018300 | glutamate dehydrogenase | 1 | Metabolism |
| [A0A6L0XMQ7](https://www.uniprot.org/uniprotkb/A0A6L0XMQ7/entry) | LINF_330011300 | hypothetical protein - conserved | 1 | Uncharacterized |
