# Supplementary material for: Functional characterization of Cullin-1-RING ubiquitin ligase (CRL1) complex in Leishmania infantum
Source: PLoS Pathog. 2024 Jul 17;20(7):e1012336. doi: 10.1371/journal.ppat.1012336 (PMC11285970; doi:10.1371/journal.ppat.1012336)
Supplement: S3 Table — For N-terminal tagging, UFP (upstream forward primer), URP (upstream reverse primer), and 5 ‘sgRNA gene-specific primers were used. For knockout, UFP, DRP (downstream reverse primer), and both 5 ‘ and 3 ‘sgRNA gene-specific primers were used. For knockout, UFP, DRP (downstream reverse primer), and both 5 ‘ and 3 ‘sgRNA gene-specific primers were used. “Upstream” and “Downstream” primers contain primer binding site compatible with pT or pPLOT plasmids (lower case letters), as well as 30 nt homology arms for recombination (uppercase letters). “sgRNA” primers consisted of a T7 RNA polymerase target sequence, 20 nt overlap with the CRISPR-Cas9 backbone sequence (lower case letters), and a 20 nt sgRNA target sequence (uppercase letters). (DOCX) [file ppat.1012336.s003.docx]

**S3 Table 1**

| ***Primers*** | **Sequence (5'- 3’)** |
| --- | --- |
| SKP-UFP-1 | CCTCTCCCCTTACTCGCTTACTCGTTGCCAgtataatgcagacctgctgc |
| SKP-URP-2 | ATCGTCGCTGTTTGAGATCTCCACCGGCATactacccgatcctgatccag |
| SKP-5sgRNA | gaaattaatacgactcactataggAAAAGTGTCAACAGCAAAGAgttttagagctagaaatagc |
| CUL-UFP-1 | AAGCGCGAAAAGGGCGTTCTTTCTTCTCCTgtataatgcagacctgctgc |
| CUL-URP-2 | GCGCAAAGCCTGCCGATCCTCTTCCAACATactacccgatcctgatccag |
| CUL-5sgRNA | gaaattaatacgactcactataggCAGCATAGAAGAAGGGAGAAgttttagagctagaaatagc |
| SKP-DRP-5 | GCAGCGGAAAAAGCACGCACACATACTCAGccaatttgagagacctgtgc |
| SKP-3sgRNA | gaaattaatacgactcactataggATAGACACTAAAGTGCATGCgttttagagctagaaatagc |
| CUL-DRP-5 | CGAGAGAGAGGCACCACGCTTCTGGCTCCGccaatttgagagacctgtgc |
| CUL-3sgRNA | gaaattaatacgactcactataggATACAAGCAGACGGGAGACAgttttagagctagaaatagc |
| RBX-UFP-1 | TGGTTGAACTGTCTCCGTAACGGATTTCCTgtataatgcagacctgctgc |
| RBX-DRP-5 | CAAAATCAAAAGCATCAAAGCGAAACTCTGccaatttgagagacctgtgc |
| RBX-5sgRNA | gaaattaatacgactcactataggGTCCTTTCTTCCGCTTCGACgttttagagctagaaatagc |
| RBX-3sgRNA | gaaattaatacgactcactataggAATATCACTTCGTCTGCAAGgttttagagctagaaatagc |
