# Supplementary material for: Functional characterization of Cullin-1-RING ubiquitin ligase (CRL1) complex in Leishmania infantum
Source: PLoS Pathog. 2024 Jul 17;20(7):e1012336. doi: 10.1371/journal.ppat.1012336 (PMC11285970; doi:10.1371/journal.ppat.1012336)
Supplement: S4 Table — (DOCX) [file ppat.1012336.s004.docx]

**S3 Table 2**

| ***Primers*** | **Sequence (5'- 3’)** |
| --- | --- |
| **SKP1 gene editing**  N-terminal F  SKP1-R  **Cullin1 gene editing**  N-terminal F  Cullin1-R | gtataatgcagacctgctgc  TAATCTCGAGCGACTCCTCGCACC  gtataatgcagacctgctgc  TACTCCATGTACAGCAGCTC |
