# Supplementary material for: Functional characterization of Cullin-1-RING ubiquitin ligase (CRL1) complex in Leishmania infantum
Source: PLoS Pathog. 2024 Jul 17;20(7):e1012336. doi: 10.1371/journal.ppat.1012336 (PMC11285970; doi:10.1371/journal.ppat.1012336)
Supplement: S5 Table — (DOCX) [file ppat.1012336.s005.docx]

**S3 Table 3**

| ***Primers*** | **Sequence (5'- 3’)** |
| --- | --- |
| **LinfSKP1**  Forw (*EcoRI*)  Rev (*XhoI*)  **LinfCUL1**  Forw (*HindIII*)  Rev (*BamHI*)  **LinfRBX1**  Forw (*EcoRI*)  Rev (*XhoI*) | ttatGAATTCatgccggtggagat  taatCTCGAGcgactcctcgcacc  taatAAGCTTatgttggaagaggatcg  tataGGATCCggcaatataatggtagacg  tataGAATTCatgcagacgaaagacg  ttaaCTCGAGagcgccatatcgaag |

* Uppercase indicates restriction enzyme sites.
