# Supplementary figures and images for: Functional characterization of Cullin-1-RING ubiquitin ligase (CRL1) complex in Leishmania infantum
Source: PLoS Pathog. 2024 Jul 17;20(7):e1012336. doi: 10.1371/journal.ppat.1012336 (PMC11285970; doi:10.1371/journal.ppat.1012336)

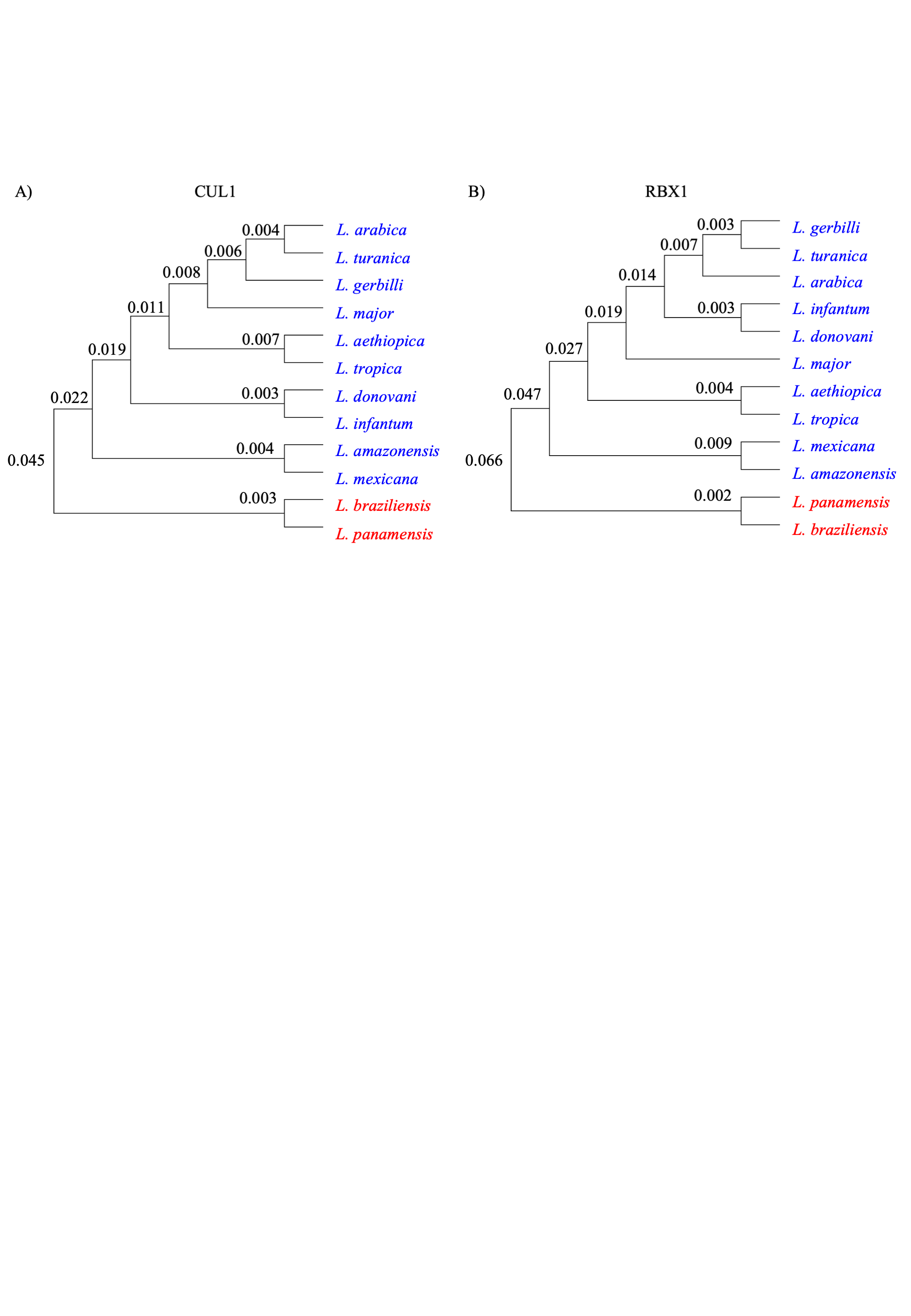

Supplement: S1 Fig — Numbers next to the branches indicated the Bayesian probability values. (TIFF) [file ppat.1012336.s006.tiff]

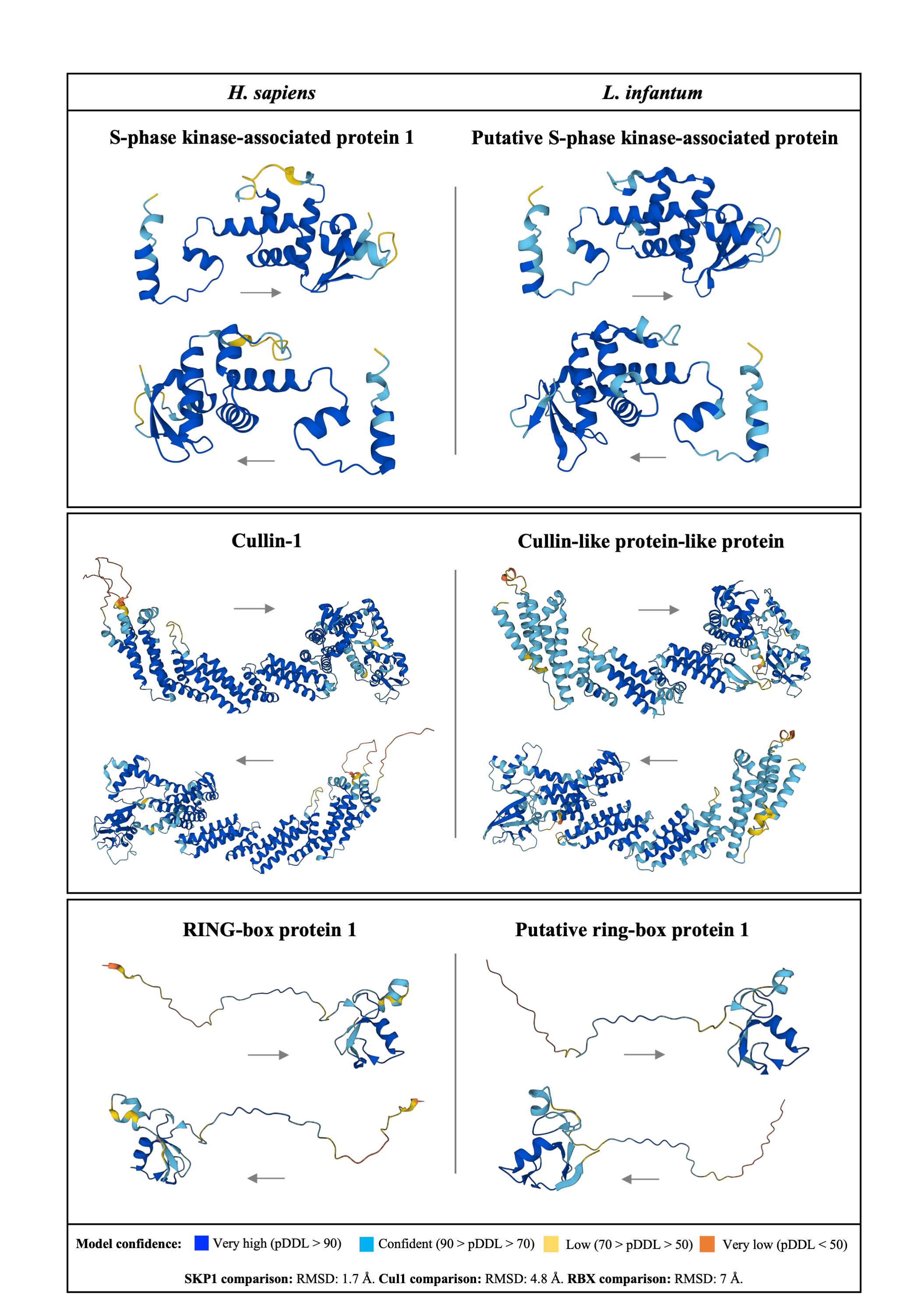

Supplement: S2 Fig — A) H. sapiens SKP1 (P63208) and L. infantum (LINF_110018100). B) H. sapiens CUL1 (Q13616) and L. infantum (LINF_240029100). C) RBX1 (P62877) and L. infantum (LINF_210005300). Three-dimensional structures were generated using Alphafold [36, 50]. The reliability scale is depicted below according to the colors represented in the structures. RMSD values indicate the similarity between the structures. (TIFF) [file ppat.1012336.s007.tiff]

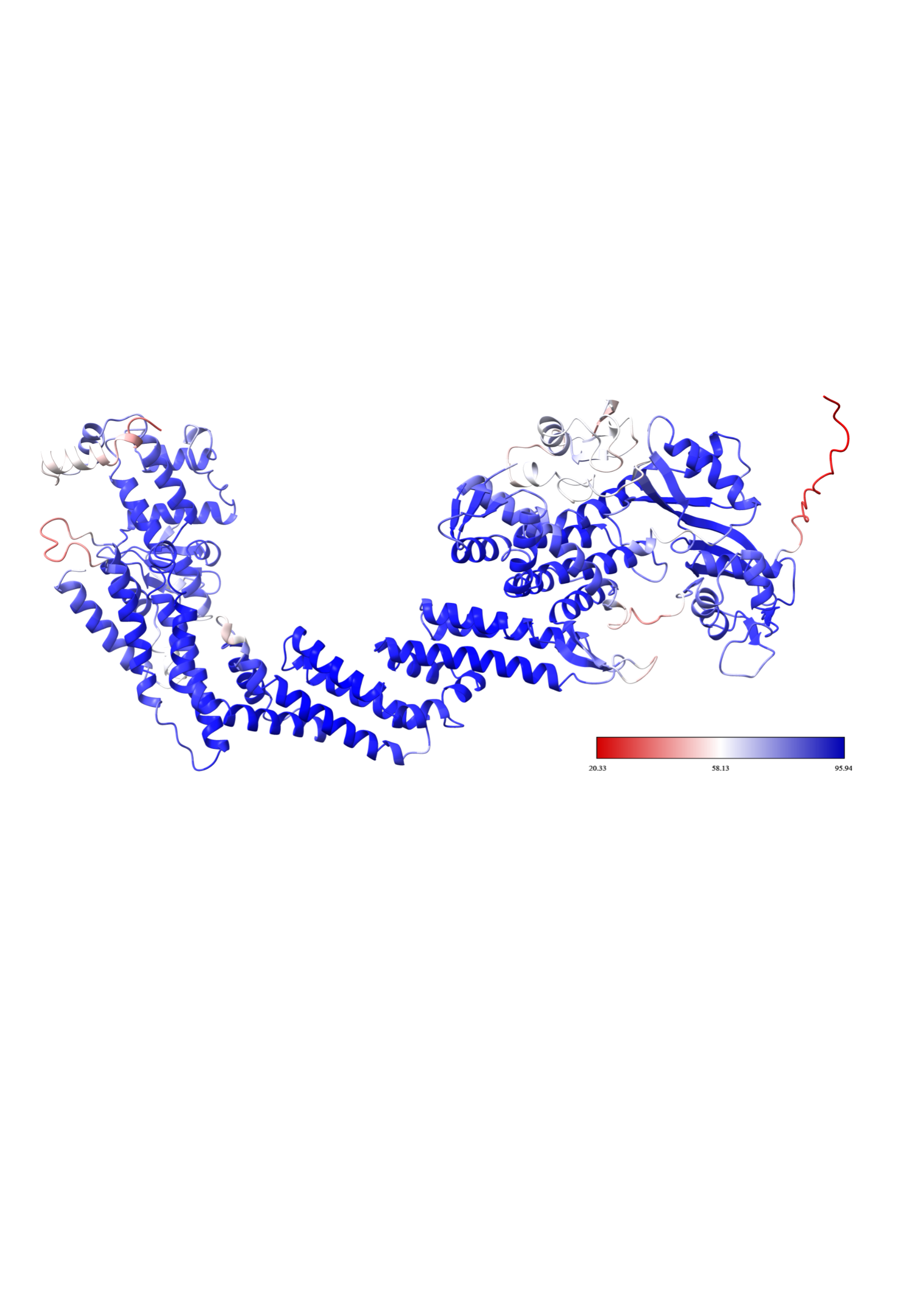

Supplement: S3 Fig — LinfSkp1, LinfCul and LinfRbx1 was used for the prediction of LinfCRL1. The model is colored by pLDDT on a red-to-blue scale, indicating an increase in the model reliability. (TIFF) [file ppat.1012336.s008.tiff]

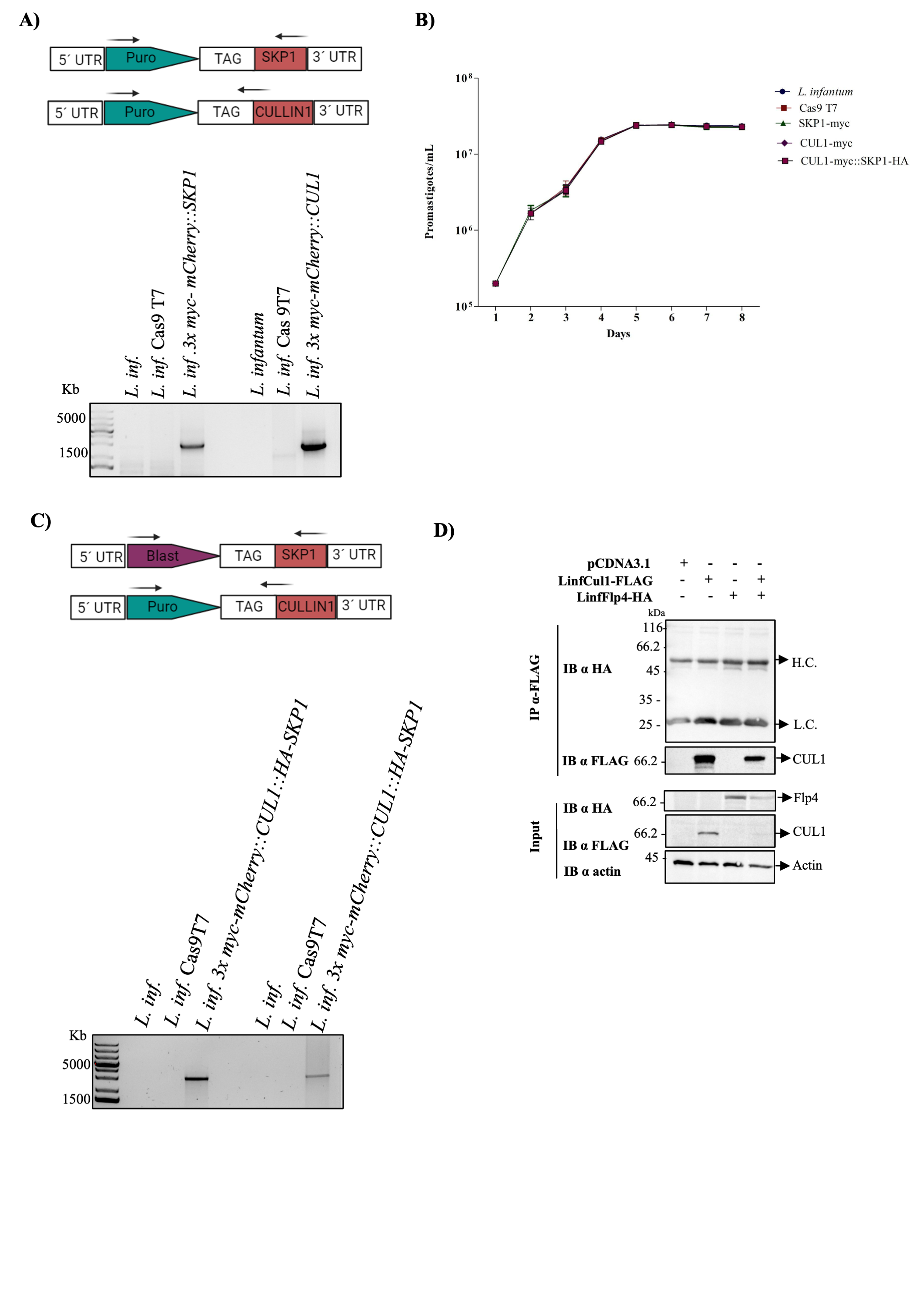

Supplement: S4 Fig — A) Diagnostic PCR for confirmation of L. infantum Cas9 T7 3x myc-mCherry::CUL1 and 3x myc-mCherry::SKP1 transgenic lines by amplification of the inserted donor DNA in the N-terminal region of SKP1 and CUL1. B) Growth curves of L. infantum Cas9 T7 3x myc-mCherry::CUL1 and 3x myc-mCherry::SKP1 compared to the L. infantum and L. infantum Cas9 T7 strains. C) Construction of the L. infantum Cas9 T7 3x myc-mCherry::CUL1::HA-SKP1 lineage. D) Immunoblotting of the interaction between LinfFlp4 and LinfCul1 in HEK293T cells. HEK293T cells were transfected with the indicated plasmids (+), and cell lysates were immunoprecipitated with anti-FLAG beads and probed with the indicated antibodies. (TIFF) [file ppat.1012336.s009.tiff]
